# Non-invasive Optical Biomarkers Distinguish and Track the Metabolic Status of Single Hematopoietic Stem Cells

**DOI:** 10.1016/j.isci.2020.100831

**Published:** 2020-01-10

**Authors:** Hao Zhou, Lisa Nguyen, Cosimo Arnesano, Yuta Ando, Manmeet Raval, Joseph T. Rodgers, Scott Fraser, Rong Lu, Keyue Shen

**Affiliations:** 1 Department of Biomedical Engineering, University of Southern California, Los Angeles, CA 90089, USA; 2 Department of Stem Cell Biology and Regenerative Medicine, University of Southern California, Los Angeles, CA 90033, USA; 3 Translational Imaging Center, University of Southern California, Los Angeles, CA 90089, USA; 4 Molecular and Computational Biology, University of Southern California, Los Angeles, CA 90089, USA; 5 Norris Comprehensive Cancer Center, University of Southern California, Los Angeles, CA 90033, USA; 6 Department of Medicine, University of Southern California, Los Angeles, CA 90033, USA; 7 USC Stem Cell, University of Southern California, Los Angeles, CA 90033, USA

**Keywords:** Optical Imaging, Biological Sciences, Cellular Physiology, Stem Cells Research

## Abstract

Metabolism is a key regulator of hematopoietic stem cell (HSC) functions. There is a lack of real-time, non-invasive approaches to evaluate metabolism in single HSCs. Using fluorescence lifetime imaging microscopy, we developed a set of metabolic optical biomarkers (MOBs) from the auto-fluorescent properties of metabolic coenzymes NAD(P)H and FAD. The MOBs revealed the enhanced glycolysis, low oxidative metabolism, and distinct mitochondrial localization of HSCs. Importantly, the fluorescence lifetime of enzyme-bound NAD(P)H (τ_bound_) can non-invasively monitor the glycolytic/lactate dehydrogenase activity in single HSCs. As a proof of concept for metabolism-based cell sorting, we further identified HSCs within the Lineage-cKit+Sca1+ (KLS) hematopoietic stem/progenitor population using MOBs and a machine-learning algorithm. Moreover, we revealed the dynamic changes of MOBs, and the association of longer τ_bound_ with enhanced glycolysis under HSC stemness-maintaining conditions during HSC culture. Our work thus provides a new paradigm to identify and track the metabolism of single HSCs non-invasively and in real time.

## Highlights

• Metabolic optical biomarkers non-invasively distinguish HSCs from early progenitors
• NAD(P)H τ_bound_ reflects lactate dehydrogenase activity in single fresh/cultured HSCs
• pHi correlates with τ_bound_ in hematopoietic populations, with HSCs being the highest
• Optical biomarkers track metabolic changes and response to drugs in cultured HSCs

## Graphical Abstract

**Figure:**
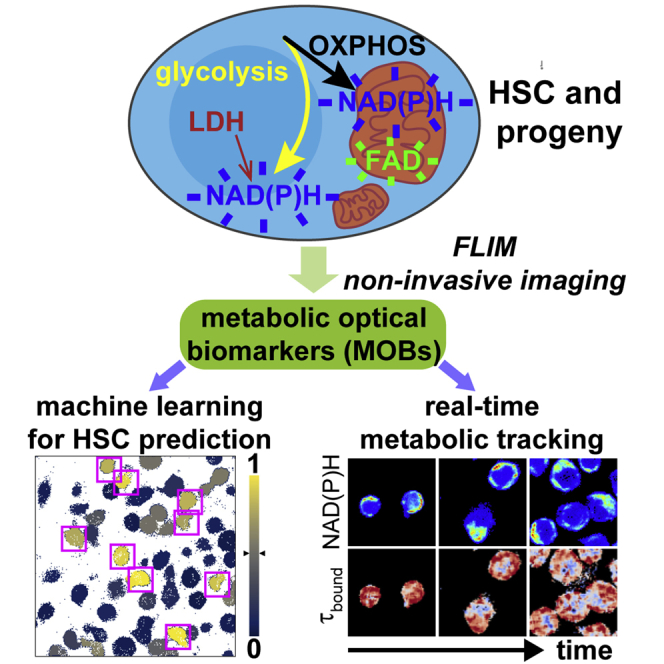

## Introduction

Hematopoietic stem cells (HSCs) can reconstitute the entire blood system and are widely used in bone marrow transplantation to treat a variety of life-threatening diseases (Wagner and Gluckman, 2010). Lately, cellular metabolism has been increasingly recognized to regulate the unique functions and the fate decisions of HSCs (Ito and Ito, 2018). Compared with the progeny, HSCs have lower oxygen consumption and prefer anaerobic glycolysis, which protects them from reactive oxygen species (ROS) (Qian et al., 2016, Simsek et al., 2010, Takubo et al., 2013) and aging (Florian et al., 2018). In contrast, mitochondrial respiration is required for HSC differentiation, and their impairment results in anemia and prenatal death (Ansó et al., 2017). Moreover, increased fatty acid oxidation promotes HSC self-renewal (Ito et al., 2012) and expansion under hematopoietic stress (Ito et al., 2016), whereas glutaminolysis supports erythroid differentiation for recovery from anemia (Oburoglu et al., 2014). Therefore, examining HSC metabolism can provide crucial information about the functional identity and fate decisions of HSCs. On the other hand, recent studies have shown that individual HSCs have different abilities to self-renew (Morita et al., 2010) and to form blood (Grinenko et al., 2014, Yamamoto et al., 2013), where metabolic heterogeneity is postulated to play a significant regulatory role through cell cycle status and ROS level (Haas et al., 2018). Indeed, HSCs with lower mitochondrial membrane potential (ΔΨ_m_) were shown to have better long-term reconstitution capacity than those with higher ΔΨ_m_ (Vannini et al., 2016). However, a more comprehensive understanding of the metabolism-function relationship has been hindered by the technical challenges of observing metabolism in HSCs at the single-cell level. Conventional bulk or destructive methods such as Seahorse assays (Ito et al., 2012) or mass spectrometry (Takubo et al., 2013) prohibit the dynamic tracking of single HSC metabolism and intact cell retrieval for *in vivo* functional studies. Most efforts on measuring single HSC metabolism have been focused on determining ΔΨ_m_ using fluorescent dyes as a surrogate for mitochondrial respiration (Kocabas et al., 2015, Simsek et al., 2010, Vannini et al., 2016, Vannini et al., 2019). However, ΔΨ_m_ provides limited information on cell metabolism, and it cannot distinguish HSCs from intermediate progenitors that share similar ΔΨ_m_ with HSCs (Simsek et al., 2010). Options are even more limited for glycolysis, a core metabolic feature and gatekeeper of HSC functions (Takubo et al., 2013), which is often measured by the uptake of fluorescent glucose analogs (Takubo et al., 2013). These chemicals do not differentiate glucose demands from different downstream metabolic pathways, compete against glucose, and may interrupt normal glycolysis (Zhu et al., 2017). All these indicators are also not suited for long-term tracking of metabolic dynamics owing to the cytotoxicity. There is thus a significant need for non-invasive, real-time approaches to assess the metabolic status of single HSCs. Addressing this need will not only enhance our ability to understand HSC heterogeneity and study their response to extrinsic/intrinsic stimuli (Haas et al., 2018), but also to monitor and preserve the quality of HSCs to improve the success rate of clinical transplantations (Watz et al., 2015) and to expand HSCs *ex vivo* to address the clinical shortages (Park et al., 2015).

**Figure 1:**
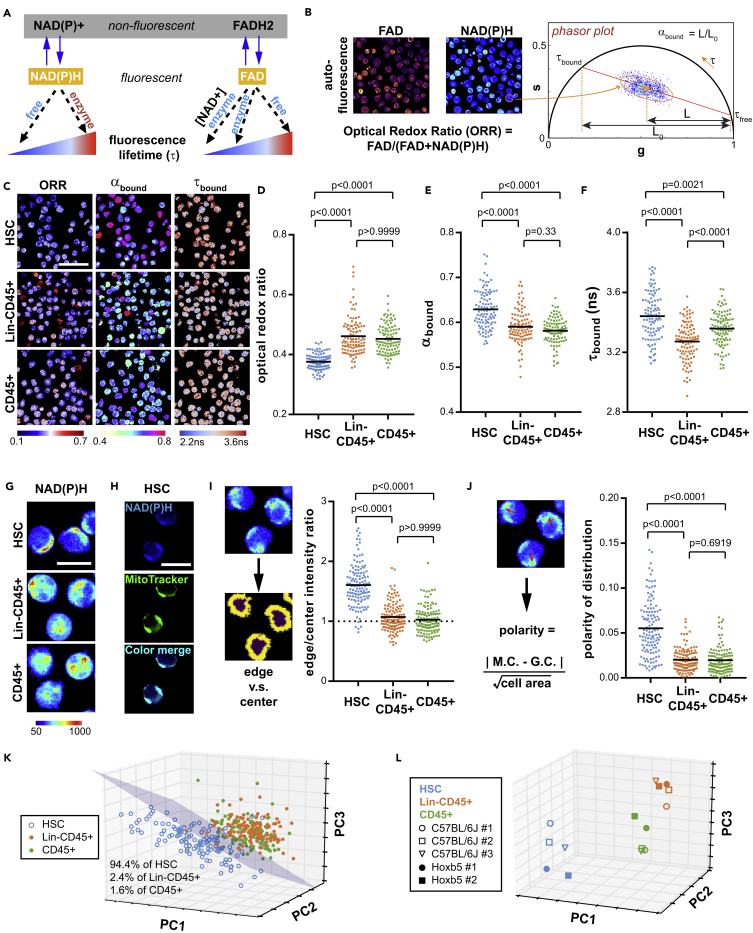
HSCs Have a Distinct Profile of Metabolic Optical Biomarkers (MOBs) at the Single-Cell and Subcellular Levels (A) Schematics of fluorescence lifetime properties of NAD(P)H and FAD. (B) Calculation of ORR (optical redox ratio), α_bound_ (ratio of enzyme-bound NAD(P)H versus total NAD(P)H) and τ_bound_ (fluorescence lifetime of enzyme-bound NAD(P)H) from single cells. (C) Representative pseudo-color images of HSCs (Lin-cKit+Sca1+Flk2-CD34-Slamf1+), Lin-CD45+ and CD45+ populations for ORR, α_bound_, and τ_bound_. Scale bar: 100 μm. (D–F) Single-cell quantification of (D) ORR, (E) α_bound_, and (F) τ_bound_ in the three populations. Each dot represents the average ORR, α_bound_ or τ_bound_ value of an individual cell. (G) Representative images of subcellular NAD(P)H distribution. Scale bar: 10 μm. (H) Pseudo-color images of NAD(P)H and mitochondria staining. Top: NAD(P)H autofluorescence signal imaged with FLIM; middle: mitochondrial staining imaged with standard confocal microscopy; bottom: color merge. Scale bar: 10 μm. (I) Ratio of NAD(P)H fluorescence intensity at the cellular edge versus center. (J) Polarity of NAD(P)H fluorescence intensity (M.C., mass center; G.C., geometric center). (K) Segregation of HSCs from the differentiated populations in a 3-D PCA plot utilizing both single-cell (ORR, α_bound_, and τ_bound_) and subcellular MOB parameters (edge/center ratio and polarity of NAD(P)H intensity). n = 127 single cells in each population. (L) Population level MOB profiles of HSCs sorted from different mice and by different markers versus Lin-CD45+ cells and CD45+ cells. Each point represents the average value of the population isolated from an individual mouse. PC, principle component. p Values: Kruskal-Wallis test. See also Figures S1–S4 and Tables S1 and S2.

Fluorescence lifetime imaging microscopy (FLIM) has been used for label-free, non-invasive observation of cellular metabolism by monitoring nicotinamide adenine dinucleotide (NADH), nicotinamide adenine dinucleotide phosphate (NADPH) and flavin adenine dinucleotide (FAD). NAD(P)H and FAD are naturally occurring auto-fluorescent metabolic coenzymes and involved in almost all metabolic pathways (Ying, 2007). Importantly, FLIM can capture the fluorescence lifetime (i.e., the characteristic time of fluorescence decay) of NAD(P)H and FAD, which changes drastically depending on their binding status with enzymes. Enzyme-bound NAD(P)H shows longer lifetime than its enzyme-free counterpart, and the balance between the two states reflect the dominant metabolic process (Lakowicz et al., 1992). Besides, the fluorescence lifetime of enzyme-bound FAD depends on the intracellular level of NAD+ (Maeda-Yorita and Aki, 1984) (Figure 1A). FLIM also allows the recording of fluorescence intensities, which reflect the quantity and distribution of the coenzymes and the redox state of cells. The intensity ratio of FAD/(FAD + NAD(P)H), known as the optical redox ratio (ORR), has been associated with the mitochondrial oxidative phosphorylation (OXPHOS) (Hou et al., 2016) and coenzyme redox states (Quinn et al., 2013) in cells. Previously, FLIM has been applied to monitor the metabolic changes in live tissues and some cancer and stem cell types (Stringari et al., 2012). Notably, FLIM-based parameters have to be interpreted under specific context since NAD(P)H participates in various metabolic pathways (Yaseen et al., 2017). Different intracellular cues, such as the types of enzyme bound to NAD(P)H, intracellular pH, and viscosity (Ogikubo et al., 2011, Plotegher et al., 2015, Vishwasrao et al., 2005) in different cellular systems can also influence FLIM readouts. Thus, applying FLIM to a specific cellular system (i.e., hematopoietic cells here) requires specific experimental validations for the interpretation of the readouts.

In this study, we aimed to establish a set of FLIM-based, non-invasive metabolic optical biomarkers (MOBs) of HSCs at the single cell level, using primary HSCs and their progeny isolated from the mouse bone marrow as a model. We achieved this by comparing HSCs against their progeny at various differentiation stages and determining the metabolic features underlying the MOBs that are unique to HSCs. We further explored the utility of these MOBs in identifying primary HSCs from the differentiated cells (including the closely related multipotent progenitors, MPPs), tracking their metabolic response to metabolic substrates and chemical drugs, as well as monitoring the real-time metabolic changes of HSCs during conditioned maintenance and expansion *in vitro*. Our study sets a foundation for identifying the biological/metabolic status of single HSCs and tracking their functions non-invasively and in real time.

## Results

### HSCs Have a Distinct Profile of Metabolic Optical Biomarkers

We used a two-photon FLIM (Browne et al., 2017) to evaluate the ability of this technique to distinguish differences in metabolic status between HSCs and the more differentiated hematopoietic cells from the bone marrow. Fluorescence-activated cell sorting (FACS) was used to sort HSCs (Lin-cKit+Sca1+Flk2-CD34-Slamf1+) (Chotinantakul and Leeanansaksiri, 2012), lineage-negative CD45-positive cells (Lin-CD45+), and CD45+ leukocytes from the bone marrow of adult mice (4–6 months old) based on their surface markers (Figure S1, Tables S1 and S2). With FLIM, we acquired fluorescence intensity and/or fluorescence lifetime images of FAD and NAD(P)H in the three populations (Figure 1B). ORR was calculated as an indicator of mitochondrial OXPHOS (Hou et al., 2016). A phasor approach was used to transform the complex multi-exponential lifetime data into 2-dimensional plots to represent fluorescence decay at each pixel of the FLIM image (Stringari et al., 2012). By averaging the clusters of pixels and determining the trajectory of pixel distribution in the phasor plot, we computed the ratio of enzyme-bound NAD(P)H versus total NAD(P)H (α_bound_) and the fluorescence lifetime values (in nanoseconds, ns) of the bound and free NAD(P)H (τ_bound_ and τ_free_) at the single-cell or image levels (Figure 1B, right; see details in Transparent Methods). HSCs had a uniformly low level of ORR compared with the Lin-CD45+ and CD45+ populations (p < 0.0001), whereas Lin-CD45+ and CD45+ cells had similar (p > 0.9999) but heterogeneous ORR levels (Figures 1C and 1D). In contrast, HSCs showed significantly higher α_bound_ of NAD(P)H, whereas the Lin-CD45+ cells were statistically indistinguishable from the CD45+ cells (Figures 1C and 1E). Moreover, HSCs had the highest τ_bound_, whereas Lin-CD45+ cells had the lowest (Figures 1C and 1F). Notably, all the three hematopoietic populations had a similar τ_free_ of approximately 0.45 ns (Figure S2A), allowing for a simplified calculation of τ_bound_ with the phasor approach in the subsequent studies (Figure S2B, see Transparent Methods).

We also identified at the subcellular level a distinct polar distribution of NAD(P)H at the edge of HSCs (Figure 1G), which is co-localized with mitochondria (Figure 1H). By treating HSC with rotenone, which inhibits mitochondrial complex I that converts NADH to NAD+ in the electron transportation chain (ETC) (Heinz et al., 2017), we confirmed a significant increase in NAD(P)H fluorescence (Figure S3A), as well as the edge/center NAD(P)H intensity ratio and polarity (Figures S3B and S3C). When segmenting individual cells into “edge” and “center” areas (Figure 1I), more than 96.0% of the HSCs exhibited accumulation of NAD(P)H at the periphery (i.e., above the edge/center intensity ratio = 1; dotted line, Figure 1I), whereas the differentiated cells had a more even distribution (54.33% of Lin-CD45+ cells and 49.61% CD45+ cells). Another spatial feature of NAD(P)H in HSCs was the asymmetric/polar distribution of NAD(P)H. To quantify this, we developed a polarity indicator, defined as the distance between the center of “mass” (NAD(P)H autofluorescence intensity) and the geometrical center of a given cell, normalized to its size (Figure 1J). The NAD(P)H autofluorescence was significantly more polarized in HSCs than in Lin-CD45+ and CD45+ populations (p < 0.0001).

Next, we investigated whether these FLIM-based parameters can distinguish the metabolic/biological status of HSCs from those of the Lin-CD45+ and CD45+ populations. We first combined the three single-cell parameters (ORR, a_bound_, τ_bound_) using principle component analysis (PCA). This was followed by linear discriminant analysis (LDA) (Li et al., 2006) to determine the segregation of the three populations. Although none of the three parameters alone could reliably distinguish HSCs, a planar gate in the PCA plot separated 80% of the HSCs (lower left side) from the majority of the other two populations (upper side of the plot; 99% of Lin-CD45+ and 98% of CD45+) (Figure S4A). Notably, by combining the two subcellular parameters (Figures 1I and 1J) with the three single-cell parameters (Figures 1D–1F), collectively termed as the metabolic optical biomarkers (MOBs), a planar gate in the 3-D PCA plot can be determined by LDA to separated ∼94% of HSCs while included only 2.4% of Lin-CD45+ and 1.6% of CD45+ cells (Figure 1K). Interestingly, the center of the three hematopoietic populations consistently appeared in the same regions of the PCA plot (Figure 1L, from five independent experiments), where HSCs were either sorted by surface markers from the wild-type mice (Figure 1L, empty symbols) or by a recently reported genotypic marker, *Hoxb5*, from a mouse model with a *Hoxb5*-tri-mCherry reporter (Chen et al., 2016) (Figure S4B; Figure 1L, filled symbols), validating the reproducibility of the results.

### Longer NAD(P)H τ_bound_ Correlates with Higher Intracellular pH and Reflects Enhanced Lactate Dehydrogenase Activity in HSCs

**Figure 2:**
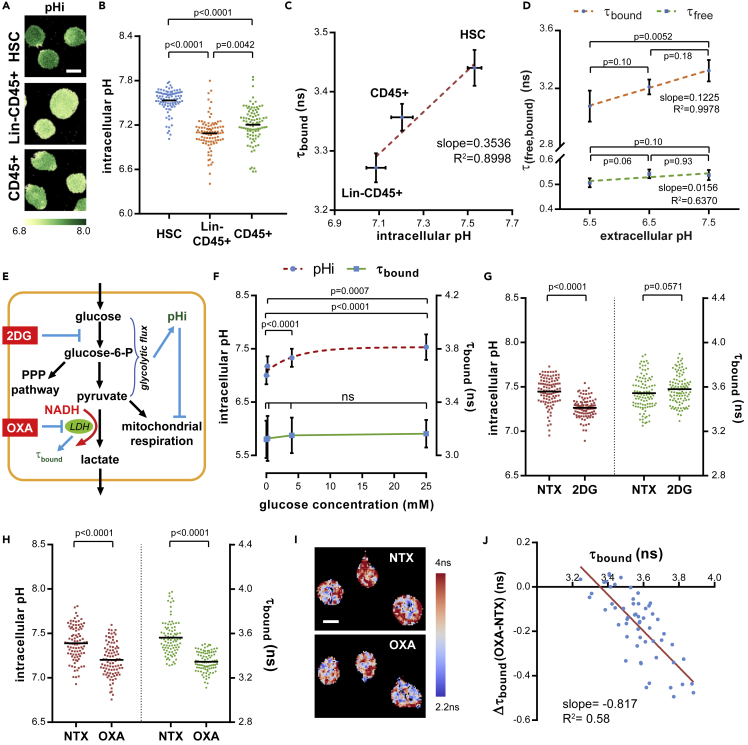
Longer NAD(P)H τ_bound_ Is Correlated with Higher Intracellular pH (pHi) in HSCs and Reflects Lactate Dehydrogenase (LDH)/Glycolytic Activity (A) Representative images of pHi in HSCs and differentiated populations. Scale bar: 5 μm. (B) Scatterplot of pHi; n = 94 single cells in each population. (C) Correlation between pHi and τ_bound_ at the population level. Error bars: standard error of the mean (SEM). (D) Correlation between NAD(P)H τ_bound_, τ_free_ in HSCs, and the extracellularly imposed pH. n = 4 images for each data point. Error bars: standard deviation (SD). p Values: ordinary one-way ANOVA. (E) Schematics of pHi and τ_bound_ regulation by glycolytic activity. (F) pHi and τ_bound_ changes in HSCs under different glucose concentration in medium. n = 16–24 cells for each condition. Error bars: standard deviation (SD). (G) pHi and τ_bound_ changes in HSCs upon 2DG treatment. n = 103 single cells for each condition. (H) pHi and τ_bound_ changes in HSCs upon OXA treatment; n = 91 single cells for each condition; p Values: Mann-Whitney test (2 conditions) or Kruskal-Wallis test (>2 conditions) for all the scatter plots above. (I) Representative pseudo-color images of τ_bound_ in individual HSC before (NTX) and after (OXA) LDH inhibition. Scale bar: 5 μm. (J) Correlation between initial τ_bound_ and Δτ_bound_ after LDH inhibition in individual HSCs. Each dot represents the average τ_bound_ and Δτ_bound_ values of a single HSC. See also Figure S5.

We next examined the metabolic or cellular functions associated with the unique MOB profile in HSCs. A distinctive feature of HSCs is their longer NAD(P)H τ_bound_. To determine what influences the τ_bound_, we examined the intracellular pH (pHi), a previously reported regulator of τ_bound_ (Ogikubo et al., 2011), in the three populations using a ratiometric pH indicator, SNARF-5F-AM (Sheldon et al., 2004). We noticed that pHi values followed a similar pattern as the τ_bound_ among the three populations, with HSCs and Lin-CD45+ cells having the highest and lowest pHi, respectively (Figures 2A and 2B). Importantly, a close linear relationship existed between pHi and τ_bound_ at the population level (R^2^ = 0.8998, Figure 2C) but not at the single cell level (R^2^ = 0.005, Figure S5A). To further examine a potential causal relationship between pHi and τ_bound_, we manipulated the pHi of HSCs maintained *in vitro* using a nigericin/K+ method (Ogikubo et al., 2011). Changing the pHi in a range between 5.5 and 7.5 did not induce significant changes in τ_free_ (Figure 2D, bottom). τ_bound_ increased in a linear relationship with the forced pHi change (Figure 2D, top, pH 5.5–7.5); however, the slope was much lower than that of the linear correlation in the three populations (0.1225 in Figure 2D versus 0.3536 ns/pH in Figure 2C). Additionally, there was no statistical difference in τ_bound_ between pH 6.5 and 7.5, indicating that pHi contributes little to the τ_bound_ differences in the physiological pH range. Previous studies have suggested a correlation between higher pHi and stem cell functions including the increased glycolysis (Webb et al., 2011), and pHi as a messenger for glycolytic flux (Dechant et al., 2010) (Figure 2E). We found that increasing glucose concentration in medium indeed enhanced the pHi (Figure 2F, top), whereas 2-deoxy-D-glucose (2DG), a glycolysis inhibitor, caused significant drop of pHi in HSCs (Figure 2G, left). Strikingly, neither the change of glucose concentration nor the addition of 2DG changed τ_bound_ (Figure 2F, bottom; Figure 2G, right), suggesting that τ_bound_ may be controlled by one or a few specific enzymes instead of all the enzymes involved in glucose metabolism. Lactate dehydrogenase (LDH) binds to NADH during pyruvate-to-lactate conversion in anaerobic glycolysis and contributes to τ_bound_ (Figure 2E). We found that inhibiting LDH by sodium oxamate (OXA) (Takubo et al., 2013), a pyruvate analog, led to significant decreases of both τ_bound_ (from 3.562 ± 0.032 to 3.345 ± 0.018 ns in Figure 2H, right) and pHi (Figure 2H, left) in HSCs. In contrast, τ_bound_ change was less significant in Lin-CD45+ and CD45+ cells (Figure S5B). Importantly, by tracking the τ_bound_ change at the single cell level, we found that the degree of τ_bound_ decrease by LDH inhibition is correlated with the initial τ_bound_ in HSCs (Figures 2I and 2J). Overall, these data suggest that the higher τ_bound_ in HSCs reflects their higher LDH activity and that τ_bound_ can be further used as a biomarker of LDH activity in individual HSCs.

### Higher NAD(P)H α_bound_ in HSCs Is Contributed by Enhanced LDH Activity

**Figure 3:**
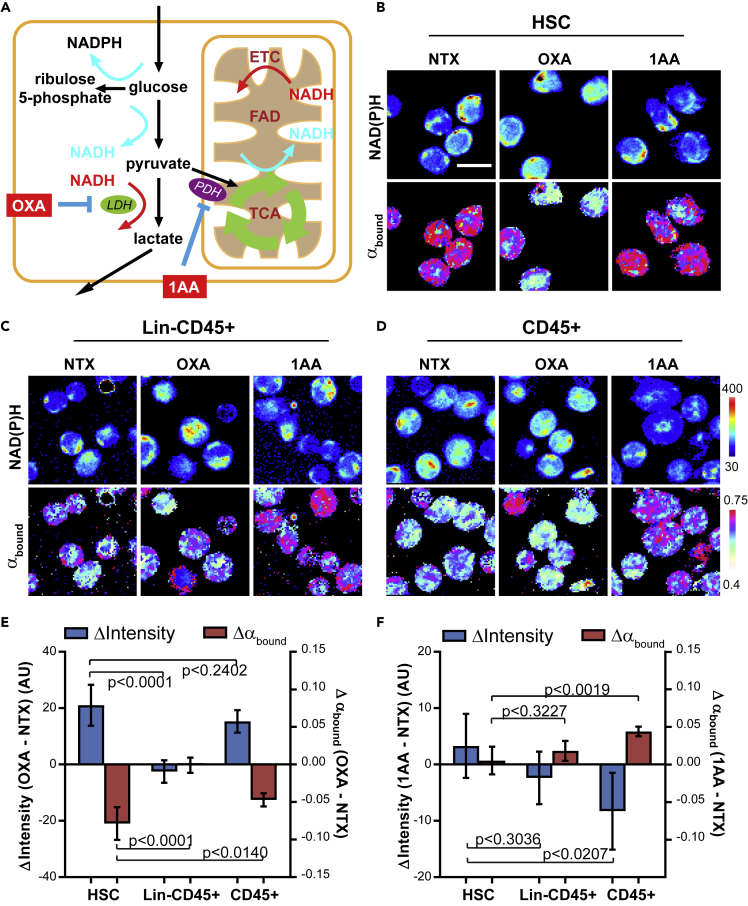
Higher NAD(P)H α_bound_ Is Contributed by LDH Activity in HSCs (A) Schematics of NAD(P)H generation (cyan arrow: released as enzyme-free form) and consumption (red arrow: consumed through enzyme-binding) in different metabolic pathways. ETC, electron transport chain; TCA, tricarboxylic acid cycle. (B–D) Representative FLIM images of NAD(P)H intensity and α_bound_ in different populations upon LDH and PDH inhibition with oxamate (OXA) and 1-aminoethylphosphinic acid (1AA), respectively. Scale bar: 10 μm. (E and F) Quantification of cellular level NAD(P)H intensity and α_bound_ changes upon LDH and PDH inhibition by OXA and 1AA, respectively. Error bars: SD. p Values: one-way ANOVA. n = 5 sets of images.

Higher α_bound_ has previously been interpreted as higher mitochondrial OXPHOS over glycolysis in FLIM studies (Stringari et al., 2012). Although the HSCs had higher α_bound_ than the more differentiated populations (Figure 1E), interpreting it as an indicator of higher OXPHOS in HSCs than their progeny is contradictory not only to the known fact that HSCs predominantly use glycolysis for energy production (Simsek et al., 2010, Takubo et al., 2013), but also to our own ORR data (Figure 1D). In the glycolytic pathway, LDH binds to NADH (which increases α_bound_) when converting pyruvate to lactate, while pyruvate dehydrogenase (PDH) releases free NADH (which decreases α_bound_) in the first step of pyruvate oxidation in mitochondria (Figure 3A). We inhibited LDH and PDH with OXA and 1-aminoethylphosphinic acid (1AA), respectively (Takubo et al., 2013). Upon OXA treatment, HSCs had a significant increase in NAD(P)H fluorescence intensity (i.e., greater accumulation of NADH) and drop of α_bound_ (i.e., decreased binding of NADH to enzymes) (Figures 3B and 3E), suggesting a large contribution of LDH activity to the high α_bound_ in HSCs. In stark contrast, OXA treatment caused little change in either NAD(P)H fluorescence intensity or α_bound_ in Lin-CD45+ cells (Figures 3C and 3E). CD45+ cells, a more complex mixture of hematopoietic cells, had an intermediate response to the treatment, between that of HSCs and Lin-CD45+ cells (Figures 3D and 3E). Interestingly, PDH inhibition induced little change in either NAD(P)H fluorescence intensity or α_bound_ in HSCs, suggesting minimal PDH activity and pyruvate shuttling into the tricarboxylic acid (TCA) cycle in HSCs (Figures 3B and 3F). In contrast, CD45+ cells showed the largest drop in NAD(P)H fluorescence intensity and the greatest increase in α_bound_ of the three populations (Figures 3D and 3F). Therefore, the higher α_bound_ in HSCs is contributed by the distinct glycolytic preference and higher LDH activity in HSCs.

### HSCs Have a Larger Pool of NADH Compared with the Differentiated Cells

**Figure 4:**
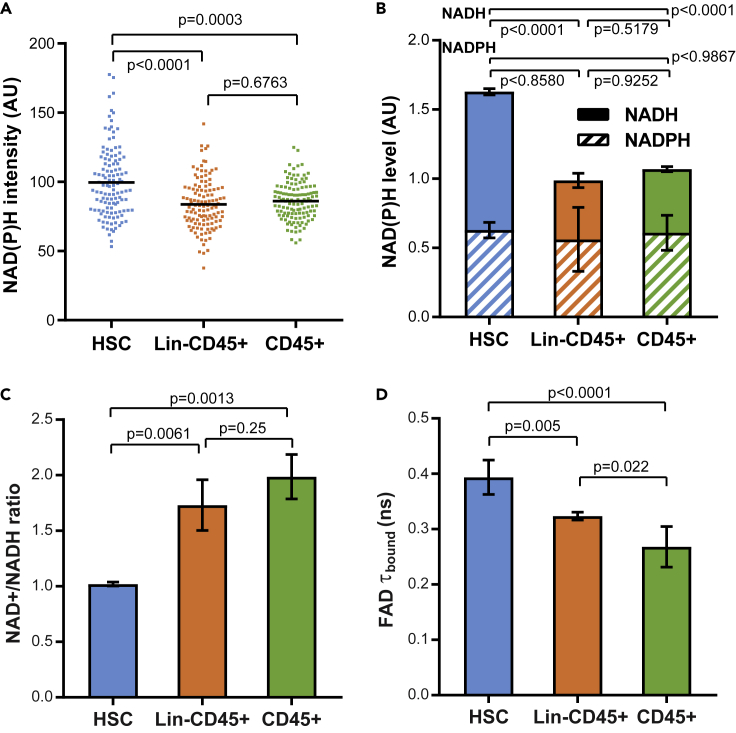
HSCs Have a More Reduced Pool of NADH (A) NAD(P)H fluorescence intensities in HSCs and differentiated cells. (B and C) (B) NADPH and NADH contents and (C) NAD+/NADH ratio measured in cell lysates (n = 3 biological replicates). (D) Fluorescence lifetime of enzyme-bound FAD in the three populations. Error bars: SD. p Values: Kruskal-Wallis test for the scatterplot; ordinary one-way ANOVA for the bar plots. See also Figures S6 and S9.

ORR is an established indicator for the relative rates of mitochondrial OXPHOS over glycolysis (Hou et al., 2016). The lower ORR in HSCs (Figure 1D) can be contributed by NADH, NADPH, or FAD, the redox states and fluorescence properties of which are intricately related to each other in cells (Ying, 2007) (Figures 1A and 1B). Measuring the autofluorescence intensities of NAD(P)H and FAD showed that the NAD(P)H level was the highest in HSCs (Figure 4A), whereas FAD signals were similar among the three populations (Figure S6). As NAD(P)H autofluorescence signal comes from both NADH and NADPH, we used a chemiluminescent assay to measure the individual NADPH and NADH levels in the lysates of the three cell types. Interestingly, the NADH level was the highest in the HSCs, whereas the NADPH levels were equivalent among the three populations (Figure 4B). Therefore, it was NADH, not NADPH, that mainly contributed to the lower ORR and larger NAD(P)H pool in HSCs. It has lately been found that ORR is proportional to the NAD+/NADH ratio, which reflects the demand for mitochondrial ATP production through NADH oxidation (Quinn et al., 2013). To validate this in HSCs, we directly measured the NAD+/NADH ratio in cell lysates using a chemiluminescent NADH assay. Consistent with the ORR data (Figure 1D), HSCs indeed showed a significantly lower NAD+/NADH ratio than the Lin-CD45+ and the CD45+ cells (Figure 4C). We further analyzed the fluorescence lifetime of enzyme-bound FAD in the three cell populations, which is negatively regulated by the NAD+ concentration through the Stern-Volmer quenching (Maeda-Yorita and Aki, 1984) (Figure 1A). At the population level, HSCs had significantly longer FAD τ_bound_ than Lin-CD45+ and CD45+ cells (Figure 4D), which agrees with the chemiluminescent measurement of NAD+. Overall, the lower ORR in HSCs reflects a larger pool of NADH and its less oxidized redox state.

### MOBs Distinguish HSCs from Multipotent and Oligopotent Hematopoietic Progenitors

**Figure 5:**
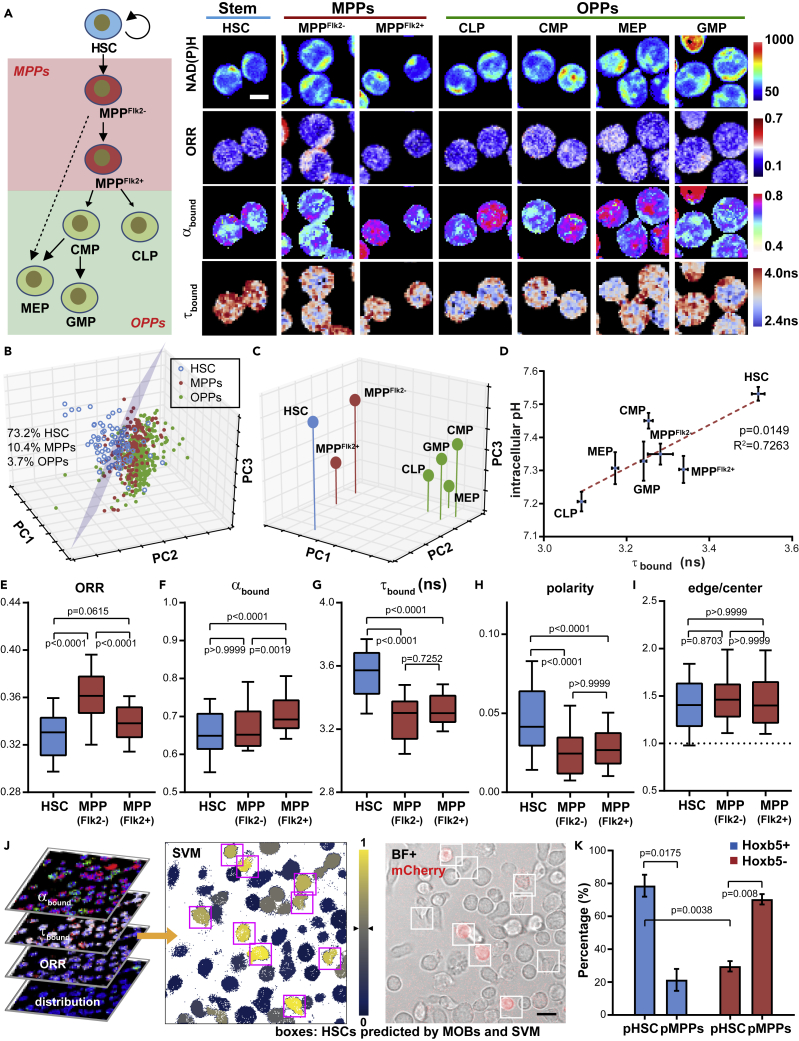
MOB Profiling Distinguishes HSCs from Hematopoietic Progenitor Cells (HPCs) (A) Representative images of NAD(P)H fluorescence intensity, ORR, α_bound_, and τ_bound_ in hematopoietic stem and progenitor cells (HSPCs). MPP, multipotent progenitor; OPP, oligopotent progenitor; CLP, common lymphoid progenitor; CMP, common myeloid progenitor; MEP, megakaryocyte/erythrocyte progenitor; GMP, granulocyte/macrophage progenitor. Scale bar: 5 μm. (B) Separation of HSCs from the MPP and OPP populations in a 3-D PCA plot using the same MOBs as in Figure 1K. (C) Metabolic shift between the center of the HSPC populations. Each dot represents the average value of a given HSPC population. n = 82 single cells in each population. (D) Correlation between pHi and τ_bound_ at the population level (τ_bound_ and pHi values are from independent experiments). n = 41 and 64 single cells for pHi and τ_bound_ in each population, respectively. Error bars: SEM. (E–I) Individual MOB parameters of HSC and MPP populations. Box plot: 10–90 percentile. p Values: Kruskal-Wallis test. (J) Left: FLIM images of KLS cells from Hoxb5-mCherry mouse to be fed into the pre-trained support vector machine (SVM) model, for the prediction of HSC and MPPs. Middle (colormap): probability of a cell being HSC based on the Platt scaling. Right: comparison of predicted HSC identity against Hoxb5-mCherry reporter expression. Scale bar: 10 μm. (K) Percentage of SVM-predicted HSCs and MPPs (pHSC and pMPPs) in Hoxb5+ (blue) and Hoxb5− (red) populations. n = 3 biological replicates. Error bars: SD. p Values: paired t test. See also Figure S7 and Tables S1 and S2.

Hematopoietic progenitor cells (HPCs), which include the multipotent progenitors (MPPs, consisting of MPP^Flk2−^ and MPP^Flk2+^) and the oligopotent progenitors (OPPs, consisting of common lymphoid progenitor [CLP], common myeloid progenitor [CMP], megakaryocyte/erythrocyte progenitor [MEP], and granulocyte/macrophage progenitor [GMP]), are rare progenitor populations downstream of HSCs in differentiation and share some similar metabolic features with HSCs via bulk measurement (Simsek et al., 2010, Takubo et al., 2013) (Figure 5A). We examined whether the above-established MOBs can also distinguish the difference between HSCs and these early progenitors. We sorted HSCs, MPPs, and OPPs based on their surface markers (Figure S1C, Table S1) and acquired FLIM images on the freshly isolated cells (Figures 5B and S7). Combining the single-cell and subcellular MOBs, we generated a 3-D PCA plot showing the difference in the MOB profile between HSCs and HPCs (MPPs and OPPs) at the single-cell resolution (Figure 5B). A planar gate could be drawn between HSCs and the HPC populations by LDA, which included the majority of HSCs (73.2%) and small fractions of MPP (10.4%) and OPP (3.7%) (Figure 5B). Interestingly, the center of the HSPC populations in the PCA plot could be visually distinguished into two groups, i.e., the early stem and progenitors (HSC and MPPs) vs. the OPPs (Figure 5C). These results show that MOBs can resolve the differences between the metabolic status of HSC and HPC populations. Notably, the linear relationship between τ_bound_ and pHi in HSCs and progenitors remained at the population level, where HSCs had the highest τ_bound_ and pHi of all the hematopoietic stem and progenitor cells (HSPCs) (Figure 5D), suggesting an increased level of anaerobic glycolysis and higher LDH activity in HSCs compared with HPCs.

MPPs are immediately downstream of HSCs in differentiation (Figure 5A). Existing protocols of HSC purification usually involve a final step of identifying long-term HSCs from MPPs through surface protein markers or efflux activities (Challen et al., 2009). Here we examined, as proof of concept, the feasibility of identifying HSCs from MPPs through the MOBs, i.e., their metabolic characteristics that are directly associated with biological functions and conserved in HSCs from animal models and humans (Kocabas et al., 2015, Simsek et al., 2010). Our initial evaluation shows that HSCs are significantly different from one or both MPPs in most of the MOBs except the edge/center NAD(P)H ratio (Figures 5E–5I). Among those, the most distinct MOBs were ORR, τ_bound_, and polarity of NAD(P)H (Figures 5E, 5G, and 5H), reflecting a less oxidative and more glycolytic and polarized phenotype of HSCs than MPPs. We then trained a support vector machine (SVM, a machine learning model) (Cortes and Vapnik, 1995) with all the MOBs from Figures 5E–5I, which predicts whether an unknown cell is an HSC based on its MOB profile (Figure 5J). To assess the predictive capacity of this model, we FACS-sorted Lin-cKit+Sca+ cells (KLS, a population composed of HSCs and MPPs) from B6 mice carrying the Hoxb5-tri-mCherry reporter, measured/analyzed the five MOBs of single KLS cells, and determined their identity through the SVM model (Figure 5J, boxed: predicted HSC, pHSC; unboxed: predicted MPPs, pMPPs). Notably, HSCs in this model can also be identified by their high expression of Hoxb5 (measured as the positivity of mCherry fluorescence, Figure 5J) through regular fluorescence microscopy (Chen et al., 2016). We then compared our prediction with the Hoxb5 expression of each cell. Importantly, our model yielded a sensitivity of 78.6 ± 7.5% (or the true positive rate, defined as the percent of Hoxb5+ cells predicted as HSCs in the Hoxb5+ cells) and a specificity of 70.4 ± 3.6% (or the true negative rate, defined as the percentage of Hoxb5− cells predicted as MPPs in all the Hoxb5− cells) from three independent experiments (Figure 5K), suggesting that MOBs can be used to directly identify HSCs in the KLS cells.

### τ_bound_ Tracks Changes in Glycolysis during *In Vitro* HSC Culture

**Figure 6:**
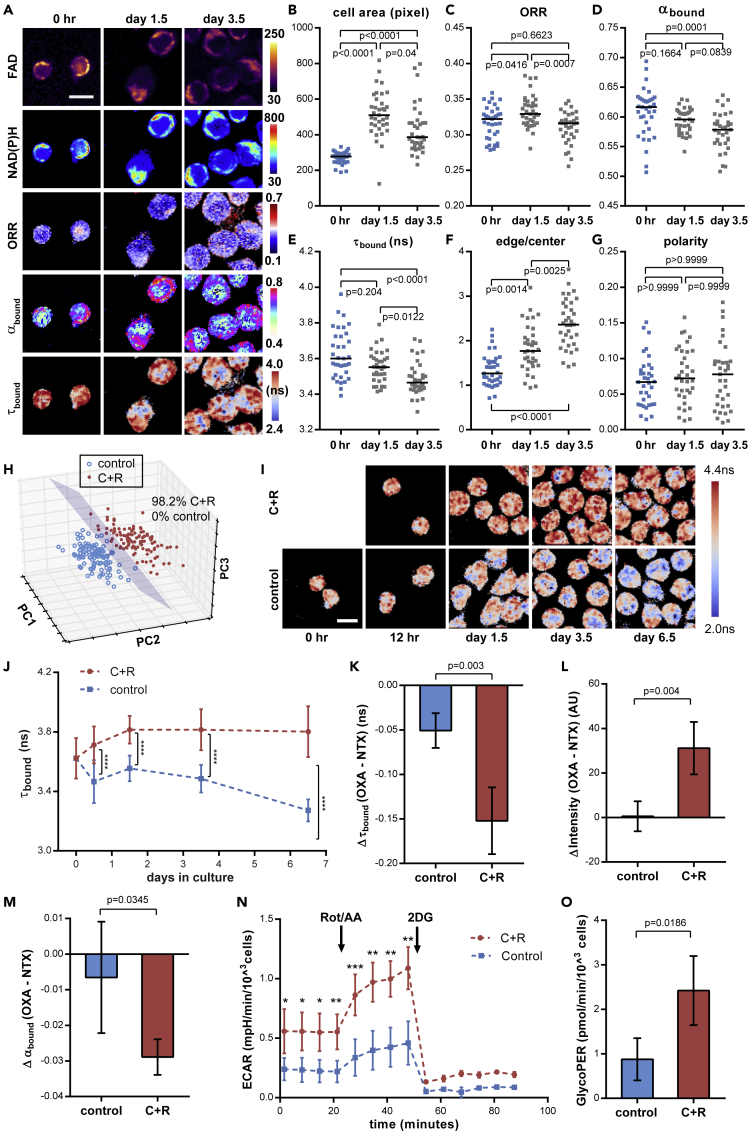
MOB Profiling and Maintenance of τ_bound_ in HSCs during *In Vitro* Culture (A) Representative images of FAD and NAD(P)H fluorescence intensity, ORR, α_bound_, and τ_bound_ of HSCs during *in vitro* culture. Scale bar: 10 μm. (B–G) Quantification of (B) cell size, (C) ORR, (D) NAD(P)H α_bound_, (E) NAD(P)H τ_bound_, (F) edge/center ratio, and (G) polarity of NAD(P)H intensity of cultured HSCs. n = 35 cells per time point. (H) 3-D PCA analysis of cultured HSCs from CHIR99021 + Rapamycin (C + R) treatment and control conditions utilizing MOBs at day 3.5; n = 113 cells per condition. (I) Representative pseudo-color images of NAD(P)H τ_bound_ in cultured HSCs over time. Scale bar: 10 μm. (J–M) (J) Quantification of τ_bound_ changes over time; n = 35 cells per condition. Quantification of NAD(P)H (K) τ_bound_ (L) intensity, and (M) α_bound_ changes of cultured HSC upon LDH inhibition at day 6.5. n = 4 sets of images. (N and O) (N) ECAR and (O) GlycoPER normalized by cell number; n = 3 biological replicates. Rot, rotenone; AA, antimycin. Error bars: SD. p Values: *p < 0.05; **p < 0.01; ***p < 0.001; ****p < 0.0001; Kruskal-Wallis test in (B-G); Mann-Whitney test in (J); t test in (K–M); paired t test in (N and O). See also Figure S8.

In normal *in vitro* cultures, loss of stemness and rapid differentiation of HSCs are accompanied by metabolic reprogramming (Liu et al., 2015). As another proof-of-concept application, we examined whether such changes can be tracked non-invasively by MOBs. We compared the freshly isolated HSCs incubated under regular cytokine condition (50 ng/mL each of SCF and TPO) against those cultured in the same medium for 1.5 and 3.5 days (Figure 6A). A significant increase in cell size was observed on day 1.5 (Figure 6B). The mean fluorescence intensities of both NAD(P)H and FAD decreased (Figures S8A and S8B), whereas the ORR slightly increased over time (Figure 6C). α_bound_ dropped continuously, suggesting a shift in the balance of metabolic pathways and/or enzyme activities (Figure 6D). Notably, τ_bound_ decreased over time, indicating a decrease of anerobic glycolysis *in vitro* (Figure 6E). The mitochondrial content of HSCs has been reported to increase during *in vitro* culture (Vannini et al., 2016). Consistently, we observed an increased accumulation of NAD(P)H at the cellular edge, resulting in a significantly higher edge/center ratio of NAD(P)H intensity at day 1.5 and 3.5 compared with the freshly isolated HSCs (Figures 6A and 6F). In contrast, the polarity of NAD(P)H autofluorescence intensity was minimally affected (Figure 6G).

Next, we investigated whether the MOBs can resolve the differences in HSC metabolism under different *in vitro* culture conditions. We treated HSCs with the Wnt activator CHIR99021 and the mTOR inhibitor rapamycin (C + R) that were previously reported to promote maintenance of HSC stemness *in vitro* when combined (Huang et al., 2012) (Figure S8C). We used cytokines (50 ng/mL each of SCF and TPO) for both conditions as the purified HSCs did not survive in the cytokine-free environment as opposed to the KLS cells (Huang et al., 2012). The C + R treatment led to a higher ORR in HSCs as a result of increased/unchanged FAD fluorescence intensity and a decrease in NAD(P)H (Figures S8D–S8F). Furthermore, both α_bound_ and τ_bound_ were significantly higher under C + R treatment (Figures S8G and S8H), whereas the edge/center ratio and the polarity of NAD(P)H fluorescence intensity showed minimal difference from the control group (Figures S8I and S8J). PCA analysis revealed that the majority of C + R-treated cells had a distinct MOB profile compared with the control group (Figure S8K: day 1.5: 98.4% versus 6.3%; Figure 6H: day 3.5: 98.2% versus 0%). As τ_bound_ is positively correlated with pHi and reflects the LDH activity in freshly isolated HSCs (Figure 2), we further monitored τ_bound_ under both culture conditions for a week. The τ_bound_ of the non-treated group dropped significantly from day 3.5, whereas it increased, then stabilized from day 1.5 after C + R treatment (Figures 6I and 6J). To examine whether the CHIR99021 or rapamycin treatment contributed to the τ_bound_ increase, HSCs were treated for 6 days with each drug separately or with the two drugs combined. CHIR99021 alone increased τ_bound_ to the same level as the C + R condition (Figure S8L). Consistent with the earlier finding of the correlation between τ_bound_ and pHi (Figures 2C and 5D), CHIR99021- and C + R-treated groups showed significantly higher pHi, whereas the rapamycin-only-treated group was statistically indistinguishable from the control group (Figure S8M). To validate whether the higher τ_bound_ in the C + R-treated group is due to enhanced glycolysis, we first inhibited LDH activities in the cultured cells under both conditions (non-treated control versus C + R) with oxamate at day 6.5. We confirmed that the τ_bound_ dropped significantly more in the C + R-treated group than in the non-treated control group (p = 0.003, Figure 6K). Moreover, C + R-treated HSCs had a much larger increase in NAD(P)H fluorescence intensity and decrease in α_bound_ than the control group (Figures 6L and 6M), indicating a release of NADH from LDH binding upon oxamate treatment. We further performed a Seahorse assay at day 6.5 to examine the glycolytic activities in the control and C + R-treated cells. Indeed, cells treated with C + R showed significantly higher extracellular acidification rate (ECAR) (Figure 6N) and glycolytic proton efflux rate (GlycoPER) (Figure 6O). Together, our results suggest that the longer τ_bound_ can serve as a biomarker for enhanced glycolysis, an important metabolic feature for HSC self-renewal *in vitro* (Guo et al., 2018).

## Discussion

Metabolic activities are key regulators and potential biomarkers of HSC functions and fates (Ito and Ito, 2018). However, to date, a major barrier to studying HSC metabolism-function relationship is the inability to non-invasively measure the glycolytic and mitochondrial metabolism in HSCs at the single-cell level. Here, we report that a set of non-invasive FLIM-based MOBs can indicate the unique high-glycolysis and low-mitochondrial OXPHOS state of single HSCs in real time. We found τ_bound_ as a biomarker for the level of LDH activity in single HSCs (Figures 2I and 2J). Importantly, high LDH activity (converting pyruvate to lactate) is characteristic of anaerobic glycolysis, a crucial metabolic feature for HSC quiescence and transplantation capacity (Takubo et al., 2013), which is previously intractable through the uptake of glucose analogs. Additionally, we can monitor the response of τ_bound_ and other MOBs to metabolic/drug treatments (Figures 2, 3, and 6), allowing for interrogating the roles of specific pathways in HSC metabolism. Moreover, a combinatorial approach was used to minimize the chance of misinterpretation and ensure the validity of our conclusion, by simultaneously monitoring several independent MOB parameters (e.g., τ_bound_, α_bound_, and NAD(P)H intensity) while introducing the extrinsic perturbations (Figures 2 and 3) and performing measurements within a short time frame upon perturbations (e.g., 1 h) by well-validated, specific inhibitors. Our work will thus set a new foundation for the study of metabolism-function relationship and heterogeneity in single HSCs. We further revealed the dynamic changes of MOBs during long-term *in vitro* HSC culture and the association of longer τ_bound_ with HSC stemness-maintaining culture conditions and enhanced glycolytic capacity. It is thus possible to study those transient but critical processes during HSC self-renewal that spans tens of hours to days, such as the apportioning and inheritance of fate determinants (Loeffler et al., 2019) related to metabolism. It may also benefit translational studies where label-free, non-invasive measurement and tracking of HSC status are needed, such as quality control for HSC transplantation (Watz et al., 2015) and long-term functional monitoring of HSCs in response to intrinsic and extrinsic stimuli *in vitro* for their biomanufacturing (Takizawa et al., 2011). Moreover, given the ability of our MOB profiling to distinguish the HSCs from the more differentiated hematopoietic progenitors, as well as to directly identify HSCs from the KLS population (Figure 5), it can potentially be used as combinatorial biomarkers for identifying HSCs in imaging cytometry and *in vivo* intravital microscopy (Ding and Morrison, 2013, Spencer et al., 2014).

NAD(P)H τ_bound_ has been reported to be influenced by several factors, including the type of coenzyme (NADH versus NADPH) (Blacker et al., 2014), the enzymes to which NAD(P)H is bound (Plotegher et al., 2015), and the pHi (Ogikubo et al., 2011). Specifically, Blacker et al. showed that τ_bound_ is positively correlated to the NADPH/NADH ratio in the HEK293 cell line, as NADPH and NADH naturally bind to different sets of enzymes involved in distinct metabolic processes (Blacker et al., 2014). However, we did not find such relationship in hematopoietic cells, as the HSC population (with the longest τ_bound_) shows a lower NADPH/NADH ratio, whereas the ratio was identical in the Lin-CD45+ and CD45+ populations with different τ_bound_ values (Figures 4B and S9). We also treated HSCs with epigallocatechin gallate (EGCG), a potent inhibitor reported to compete for NADPH but not NADH binding sites (Blacker et al., 2014), but did not observe a significant difference as seen in HEK293 cells by Blacker et al. (Figure S10). Therefore, τ_bound_ does not seem to correlate with the NADPH fraction in hematopoietic cells, likely owing to the differences in metabolic programs between hematopoietic populations and HEK293 cells. Ogikubo et al. reported in HeLa cells that τ_bound_ varies with changes in pHi in a negative correlation (Ogikubo et al., 2011). In contrast, we found a positive linear correlation between τ_bound_ and pHi at the population level in hematopoietic cells, as well as in the pHi perturbation experiments (Figures 2C and 2D). We found that the pHi of HSCs was the highest among all the hematopoietic populations tested in the study, which was contributed by both glycolysis and LDH activity (Figures 2F–2H). It is noteworthy that higher pHi has been observed in mesenchymal stem cells (MSCs) (Meleshina et al., 2018), human induced pluripotent stem cells (hiPSCs) (Chao et al., 2018), and cancer cells (Webb et al., 2011). It has been reported to associate with higher glycolysis (Lindström and Sehlin, 1984) and inhibition of mitochondrial activity and cell cycle (da Veiga Moreira et al., 2015). The higher pHi and longer τ_bound_ may thus be common features and potential biomarkers in other quiescent stem cells that rely on glycolysis (Pala et al., 2018). Interestingly, disturbing glycolysis with 2DG decreased pHi but not τ_bound_ in HSCs. This is possibly due to the reduction of the whole glucose metabolism by 2DG, which may proportionally impact the enzyme binding of NAD(P)H in all the downstream pathways such as lactate production, pentose phosphate pathway (PPP), and mitochondrial respiration (Figure 2E), resulting in an unchanged overall τ_bound_. With LDH inhibitor oxamate, we confirmed that τ_bound_ reflects the relative level of LDH activity at the single cell level. We also showed that LDH inhibition led to lower pHi, likely as a result of reduced glycolysis (Dechant et al., 2010) consistent with the 2DG inhibition. Another potential contributing mechanism of LDH activity to the higher pHi is the consumption of protons during the pyruvate-to-lactate conversion (Acharya et al., 2014). However, we did not observe a significant correlation between τ_bound_ and pHi at the single-cell level (Figure S5A), suggesting that LDH activity and its associated proton consumption may not be the primary determinant of the pHi level.

The NAD(P)H α_bound_ is among the single-cell parameters that distinguish HSCs from Lin-CD45+ and CD45+ cells, as it is significantly higher in HSCs. Although α_bound_ has been previously considered as an indicator of mitochondrial OXPHOS over glycolysis (Stringari et al., 2012), we have shown here that LDH-NADH binding activities during anaerobic glycolysis contributed to the high α_bound_ in HSCs (Figures 3B and 3E). We also showed through the response of NAD(P)H intensity and α_bound_ to 1-AA treatment that HSCs have minimal PDH activity in mitochondria (Figures 3B and 3F), consistent with a previous observation that PDH is suppressed in HSCs through phosphorylation by PDH kinase (Takubo et al., 2013). Our results here thus provide an alternative interpretation for higher α_bound_ in future FLIM-based cell studies. Notably, it was shown in the previous report that HSCs incubated with 1-AA have more pyruvate accumulation than the control group after 4 days of *in vitro* culture (Takubo et al., 2013). Since HSCs differentiate rapidly during *in vitro* culture (Vannini et al., 2016), the untreated control were likely diluted by the differentiated cells after 4 days. Thus, these data suggest that the better maintained HSCs (by 1-AA) have less PDH activity than the more differentiated cells, which agrees with our observation.

The cellular redox state is known to play an important role in regulating cell fate and functions. Our results show that HSCs have a low ORR and NAD+/NADH ratio, indicating a less oxidized pool of NADH (Figures 1D and 4C). Moreover, we also observed a lower NADPH/NADH ratio in HSCs (Figure S9). As the intracellular redox state reflects the balance between oxidizing and reducing agents/pathways, the lower NADPH/NADH ratio may suggest a lower demand for reducing agent and/or lower oxidative stress in dormant HSCs. Our results and others suggest that the reduced redox state can be a consequence of a low environmental oxygen level in the HSC hypoxic niche (Simsek et al., 2010, Spencer et al., 2014). Interestingly, in agreement with our findings, the NAD+/NADH ratio has also been reported to be negatively regulated by pHi (Orij et al., 2009). Moreover, NAD+/NADH can regulate enzymatic activities as the activity of PDH, which initiates mitochondrial respiration, is inhibited by a low NAD+/NADH ratio (Berg et al., 2002).

In addition to the single-cell parameters, we report here a peripheral and polarized distribution of NAD(P)H autofluorescence in HSCs at the subcellular level. The unique spatial NAD(P)H distribution overlaps with mitochondria in HSCs (Figure 1H), similar to what was observed previously in other cell types (Dölle et al., 2010). Our results suggest a peripheral distribution of mitochondria, which may be related to HSC functions such as mitochondrial quality control (Filippi and Ghaffari, 2019) and transferring mitochondria to stromal cells (Golan et al., 2016). The nucleus is located in the central dim region of cells (Blacker et al., 2014). Thus, the relatively low NAD(P)H intensity in the nucleus can be a result of less active biosynthesis in HSCs (Ying, 2007). Polarity of cellular components is known to regulate stem cell functions and fate, and asymmetrical apportioning of aged mitochondria in stem-like cells is required to maintain stemness in one of the daughter cells (Katajisto et al., 2015). The increase in the edge/center ratio and polarity of NAD(P)H intensity upon rotenone treatment suggests that the polarized distribution may reflect spatially uneven metabolic activities in HSCs (Figure S3). Tracking the redistribution of NAD(P)H during cell division will thus potentially reveal the metabolic phenotypes of daughter cells and whether the metabolic asymmetry plays a role in regulating HSC self-renewal.

Using MOBs, we have further shown that the MOB profile can distinguish the unique metabolic/biological status of HSCs from their downstream early progenitors (Figures 5A–5C). Importantly, there remains a positive correlation between τ_bound_ and pHi among HSPC populations (Figure 5D), where both τ_bound_ and pHi are longer and higher in HSCs than in progenitors. Interestingly, CLP and CMP, the two daughter populations derived directly from MPPs, demonstrate dramatically different τ_bound_ and pHi (Figures 5A and 5D). These results suggest that glycolysis may play an important role in cell fate decisions and lineage commitment during early hematopoiesis. We observed a distinctly higher ORR in MPP^Flk2−^ and MEP compared with most HSPCs (Figure S7A), which corroborates with the report that MPP^Flk2−^ directly gives rise to MEP (Chotinantakul and Leeanansaksiri, 2012). Importantly, as a proof-of-concept application, a combination of MOBs with machine learning can be used to identify HSCs from the commonly enriched KLS cells. Notably, some groups have tried enriching HSCs based on the metabolic characteristics, such as the low ROS (Jang and Sharkis, 2007) or ΔΨ_m_ (Simsek et al., 2010) levels of HSCs. In addition to the potential cytotoxicity of the invasive dyes used in these studies, it is known that HSCs can extrude dyes through efflux activities (Goodell et al., 1996), which complicates the metabolic interpretation of these results. Verapamil, an efflux inhibitor commonly used for mitochondrial staining, was lately shown to influence the readouts of ΔΨ_m_ and mitochondrial mass (Bonora et al., 2018) and inhibit glycolysis (Strigun et al., 2011). Moreover, it was shown that the ROS^low^ and ROS^high^ subsets from Lin-CD45+ population contain equal number of phenotypic HSCs and progenitor cells (Jang and Sharkis, 2007), suggesting that ROS level alone cannot enrich the phenotypic HSCs or distinguish them from progenitors. Considering those points, our approach is advantageous as it is label-free, reflects the distinct metabolic processes/features, and can distinguish HSCs directly from MPPs.

The difficulty to maintain HSC stemness *in vitro* has been linked to the loss of physiological niche conditions and consequent metabolic reprogramming (Huang et al., 2012, Liu et al., 2015). However, this metabolic change has barely been studied in single HSCs in a non-invasive, quantitative, and real-time manner. As a proof of concept, we monitored the changes in the FLIM-based metabolic profile of HSCs *in vitro* and under pharmacological treatments. We observed a significant increase in cell size and decrease in the mean NAD(P)H fluorescence intensity over a prolonged period of culture (Figure S8A), possibly due to the entrance of cell cycle and rapid proliferation. Most of the MOBs in cultured cells, including ORR, α_bound_, τ_bound_, and edge/center NAD(P)H intensity ratio, became different from those in the freshly isolated cells, reflecting a traceable metabolic shift over time *in vitro*. These results suggest a rapid switch in metabolic pathways, redox state, and co-enzyme redistribution and a quick adaptation of cellular metabolism to the new environment during *in vitro* HSC culture. Strikingly, under pharmacologic treatment that promotes maintenance of HSC stemness *in vitro* (Huang et al., 2012), 98.2% of HSCs were metabolically distinct from the non-treated cells at day 3.5 (Figure 6H), which underscores the sensitivity of the MOB profile to external stimuli in *ex vivo* HSC cultures. As τ_bound_ is independent from NAD(P)H level and sensitive to glycolysis and LDH activity, we utilized it to track the glycolytic level of HSCs *in vitro*. Our results show that HSCs lost their distinctive, longer τ_bound_ under normal culture conditions; in contrast, they had further increased τ_bound_ under the pharmacologic treatment. Importantly, consistent with our metabolic interpretation of longer τ_bound_, Seahorse assay and LDH-specific inhibition study confirmed the enhanced glycolytic functions under pharmacologic treatment (Figures 6K–6O). Notably, the Wnt activator CHIR 99021, but not the mTOR inhibitor Rapamycin, led to the same enhanced τ_bound_ under the combination of both drugs. Interestingly, other Wnt activators, including prostaglandin E2 (PGE2) and StemRegenin 1 (SR-1), have also been shown to enhance HSC maintenance and expansion (Goessling et al., 2009, Wagner et al., 2016). Importantly, Wnt pathway has lately been found to promote pyruvate dehydrogenase kinase (PDK) activity to enhance glycolysis and inhibit mitochondrial OXPHOS in colon cancer (Pate et al., 2014). Additional studies are needed to further investigate the signaling pathway between Wnt activation to glycolysis in HSCs. Consistent with longer τ_bound_, higher pHi was also observed under stemness-promoting treatment. Overall, our data support τ_bound_ as a biomarker of glycolysis of HSCs *in vitro*.

In this study, we demonstrate the ability of label-free FLIM imaging and a set of non-invasive MOB parameters to identify and track the metabolic properties of single HSCs, to address an unmet need in HSC research. There remain challenges in precisely interpreting the MOB parameters, as NAD(P)H signal includes both NADPH and NADH, whereas both NAD(P)H and FAD are involved in multiple metabolic pathways. More mechanistic studies are thus still needed to elucidate what regulates these metabolic characteristics. Nevertheless, our study sets a foundation for *in vivo* studies on metabolism-function relations and heterogeneity in HSCs when combined with single-cell handling techniques (Gross et al., 2015). It also allows for metabolic studies of HSCs *in vitro* and discovery of suitable conditions for HSC *ex vivo* maintenance and/or expansion. Physiological and pathological processes, including self-renewal (Morita et al., 2010), differentiation (Grinenko et al., 2014, Yamamoto et al., 2013), aging (de Haan and Lazare, 2018), inflammation (Pietras, 2017), and hematological diseases/malignancies (Mihaylova et al., 2014), can be further studied using these established biomarkers in real time. Moreover, given the advantages of two-photon microscopy, such as deep tissue penetration, minimized light scattering in tissue, low background signal level, and low photobleaching, this methodology can potentially be adapted for *in vivo* HSC study through intravital imaging (Spencer et al., 2014). Our findings may also be extended to human HSCs, which share metabolic similarities with murine HSCs (Guo et al., 2018) but have a different set of surface markers (Chao et al., 2008).

### Limitations of the Study

In the present study, we used a set of MOBs derived from the fluorescent properties of NAD(P)H and FAD to distinguish and monitor the metabolic features/status of single HSCs non-invasively and in real time. However, given the fact that NAD(P)H and FAD participate in almost all the metabolic pathways, it remains difficult to precisely deconvolute the contributions of different pathways and their changes. Another limitation is that NADH and NADPH are spectrally indistinguishable; it is therefore technically challenging to further differentiate the contribution of NADH and NADPH in MOBs and cellular metabolism. Future incorporation of more direct, invasive single-cell measurements (such as those being developed with mass spectrometry) (Yin et al., 2019) will allow for more definitive validation or elucidation of the contributing elements at the single-cell level. In this study, we used the Hoxb5 model for the proof-of-concept validation of the MOBs and machine learning-based prediction of HSCs. Although the model has been characterized for the enrichment of HSCs, there is still a lack of published data on more detailed characteristics of Hoxb5+ cells in different hematopoietic subpopulations in bone marrow. *In vivo* multilineage reconstitution assays will thus be a more definitive route to validate the MOBs- and machine learning-predicted HSCs. On the other hand, further development of techniques and platforms to isolate cells based on their MOB profiles will be needed to perform such *in vivo* functional analysis. Future studies will further focus on elucidating the mechanisms that dictate why stem and progenitor/differentiated cells differ in their metabolic parameters.

## Methods

All methods can be found in the accompanying Transparent Methods supplemental file.

## Data and Code Availability

The datasets generated during and/or analyzed during the current study are available from the corresponding author on reasonable request.

## Supplemental Information

Document S1. Transparent Methods, Figures S1–S10, and Tables S1 and S2
