# Supplementary material for: Non-invasive Optical Biomarkers Distinguish and Track the Metabolic Status of Single Hematopoietic Stem Cells
Source: iScience. 2020 Jan 10;23(2):100831. doi: 10.1016/j.isci.2020.100831 (PMC6994633; doi:10.1016/j.isci.2020.100831)
Supplement: Document S1. Transparent Methods, Figures S1–S10, and Tables S1 and S2 [file mmc1.pdf]

iScience, Volume *23*

Supplemental Information

Non-invasive Optical Biomarkers Distinguish and Track the Metabolic Status of Single Hematopoietic Stem Cells

Hao Zhou, Lisa Nguyen, Cosimo Arnesano, Yuta Ando, Manmeet Raval, Joseph T. Rodgers, Scott Fraser, Rong Lu, and Keyue Shen

Supplemental Information

Supplemental Figures S1-S9
Supplemental Tables S1-S2
Transparent Methods
Supplemental References

Supplemental Figures

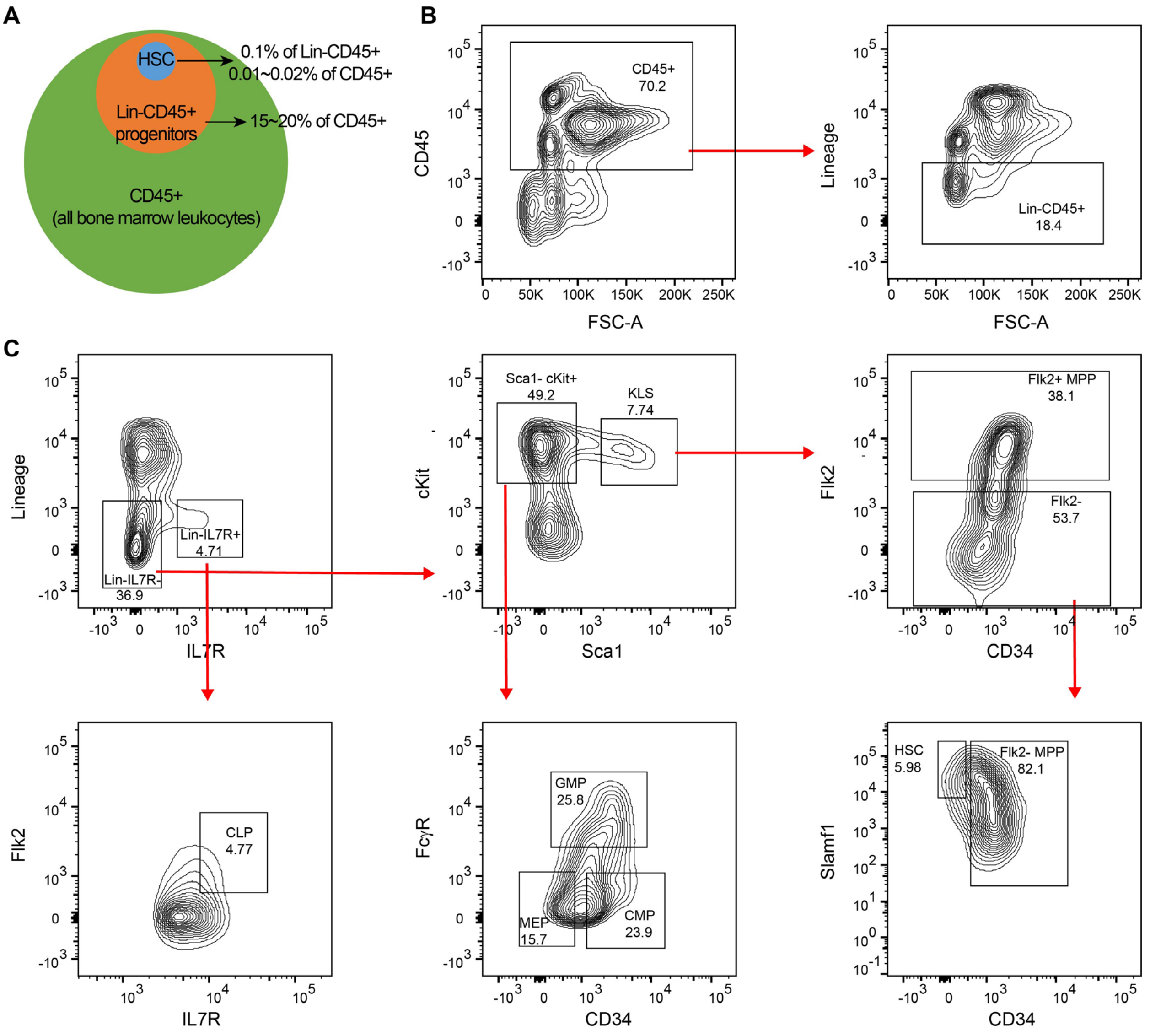

Figure S1. *Gating for hematopoietic populations harvested from bone marrow*. **Related to Figure 1 and Figure 5.** (A) The relationship of three sorted populations (HSC, Lin-CD45+, and CD45+) from the bone marrow; (B) Gating scheme for CD45+ and Lin-CD45+ populations; (C) Gating scheme for HSCs, multipotent progenitors (MPPs), and oligopotent progenitors (OPPs). KLS: cKit+Lin-Sca1+; CLP: common lymphoid progenitor; CMP: common myeloid progenitor; GMP: granulocyte/macrophage progenitor; MEP: megakaryocyte/erythrocyte progenitor. Sorting starts from DAPI singlets. Numbers indicate the percentage of the gated populations to the parent population.

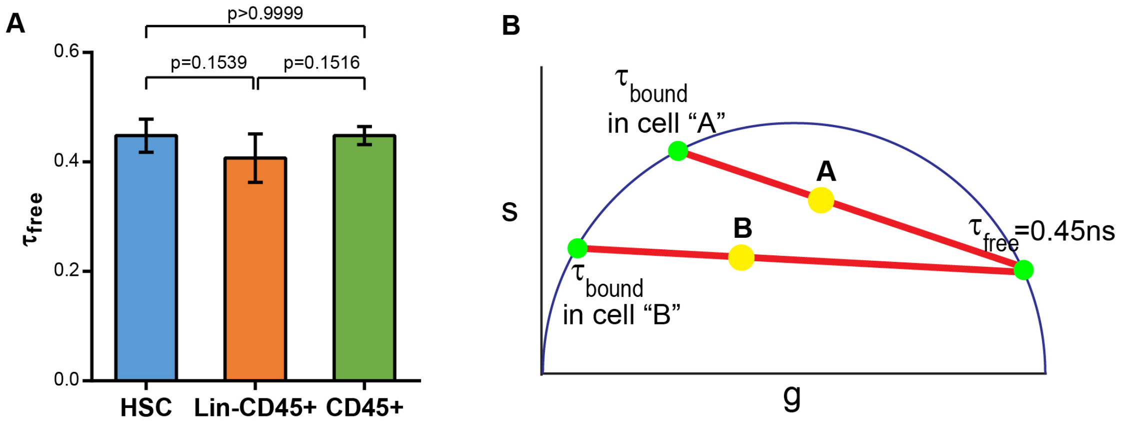

Figure S2. *Calculation of τ_{bound} in individual cells.* **Related to Figure 1**. (A) τ_{free} in different populations, calculated by 95% confidence ellipse fitting all the pixels from the cells in each image (Methods); n = 4 pictures for each population. Error bars: standard deviation. Error bars: SD. P values: ordinary one-way ANOVA. (B) Schematics of τ_{bound} calculation in individual cells using the phasor plot.

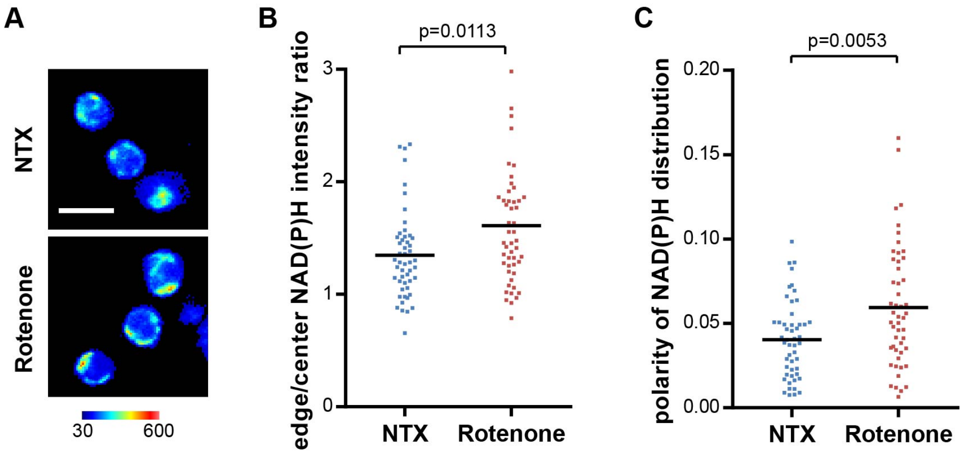

Figure S3. *Localization of NAD(P)H signal with mitochondria.* **Related to Figure 1**. (A) Representative images of NAD(P)H intensity changes upon rotenone treatment; Rotenone treatment increased (B) edge/center ratio of NAD(P)H fluorescence intensity; and (C) polarity of NAD(P)H distribution. n = 50 single cells in each group in (B,C). Scale bar: 10 μm; P-values: Mann-Whitney test.

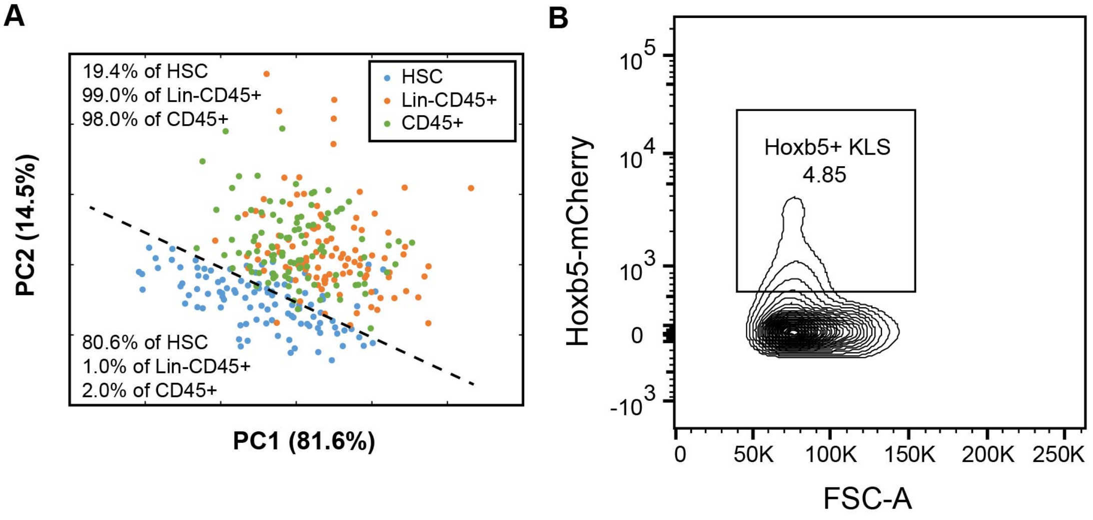

Figure S4. *Segregation of HSCs from differentiated cells.* **Related to Figure 1**. (A) The segregation was shown utilizing single-cell MOBs (ORR, α_{bound} and τ_{bound}) in a 2-D PCA space. Dataset is the same as in **Fig. 1D-F**. (B) Gating scheme for HSCs with Hoxb5-mCherry marker (parent population: cKit+Lin-Sca1+ (KLS) cells).

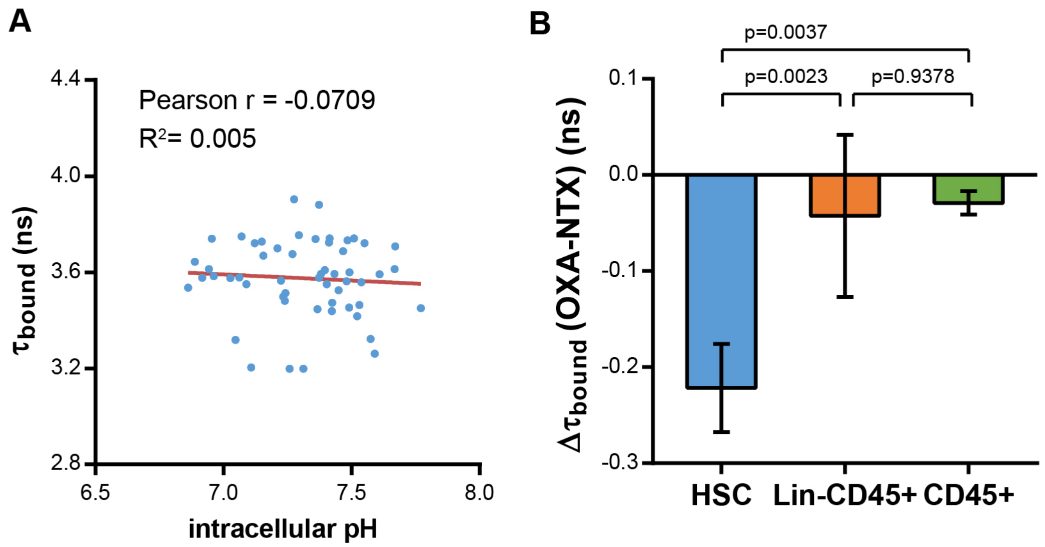

Figure S5. *Correlation between pHi, NAD(P)H τ_{bound} and LDH activity.* **Related to Figure 2**. (A) Correlation between pHi and τ_{bound} in HSCs at the single cell level. n=54 single cells. (B) Decrease of τ_{bound} in different populations upon oxamate treatment. n = 5 sets of pictures. Error bars: SD. P-values: ordinary one-way ANOVA.

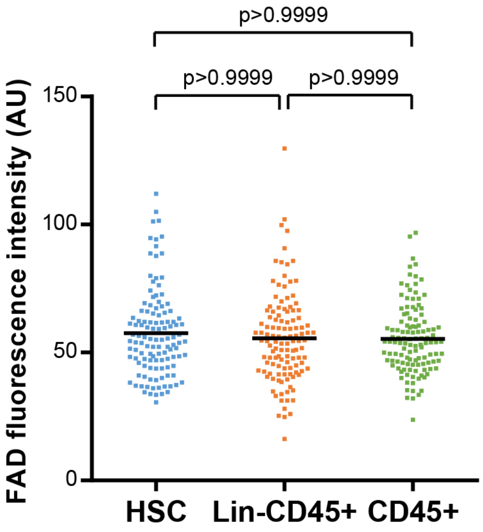

Figure S6. *Similar FAD fluorescence in HSCs and differentiated cells.* **Related to Figure 4**. Dataset is the same as in **Figure 4A**. P-values: Kruskal-Wallis test.

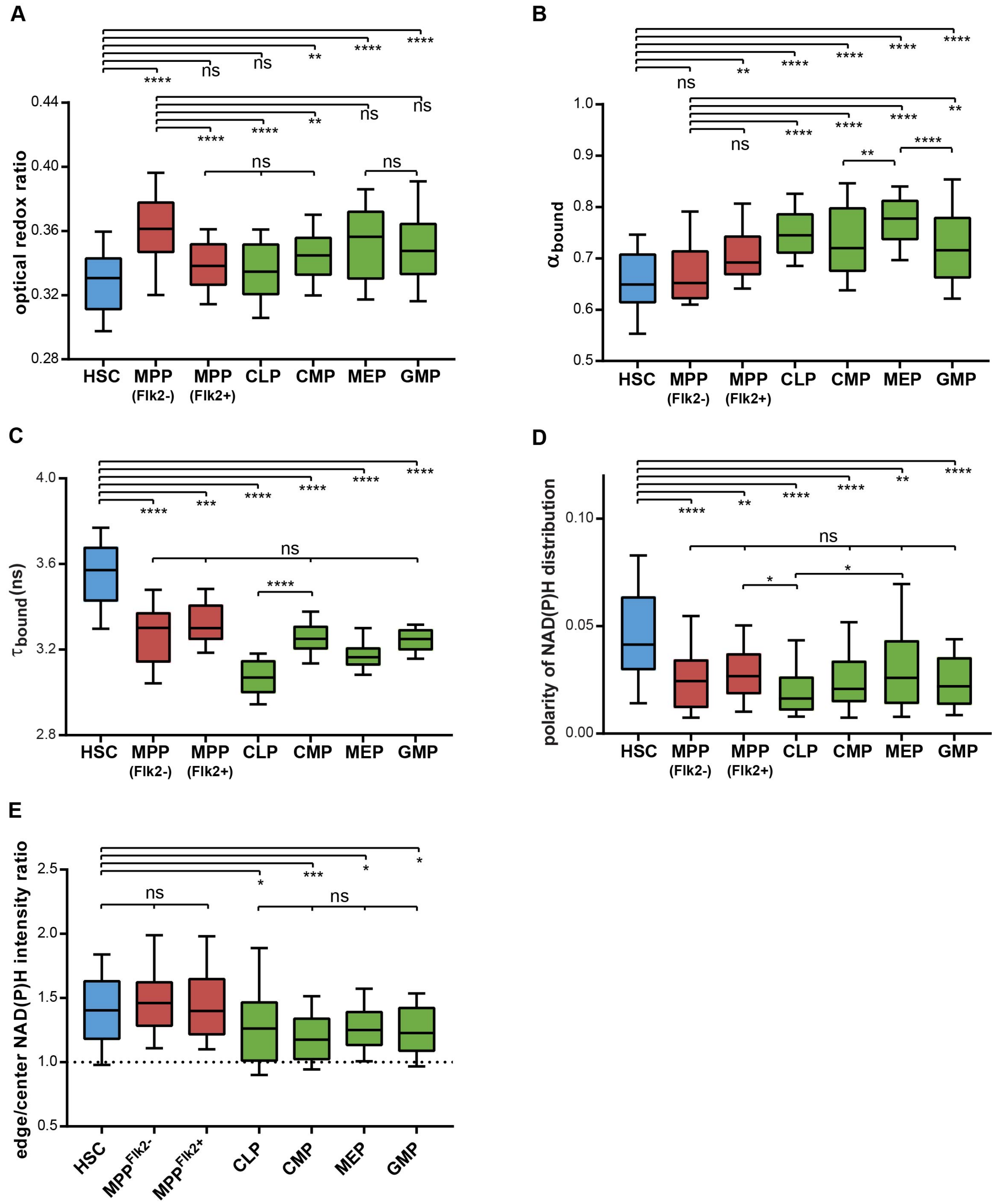

Figure S7. *HSCs have a MOB profile distinct from those of multipotent and oligopotent progenitors*. **Related to Figure 5.** Box plot of (A) ORR; (B) α_{bound}; (C) τ_{bound}; (D) polarity and (E) edge/center ratio of NAD(P)H. Box plots: 10–90 percentile. n = 80 cells for each population. P values: n.s., p > 0.05; *p < 0.05; **p < 0.01; ***p < 0.001; ****p < 0.0001; analyzed with Kruskal-Wallis test.

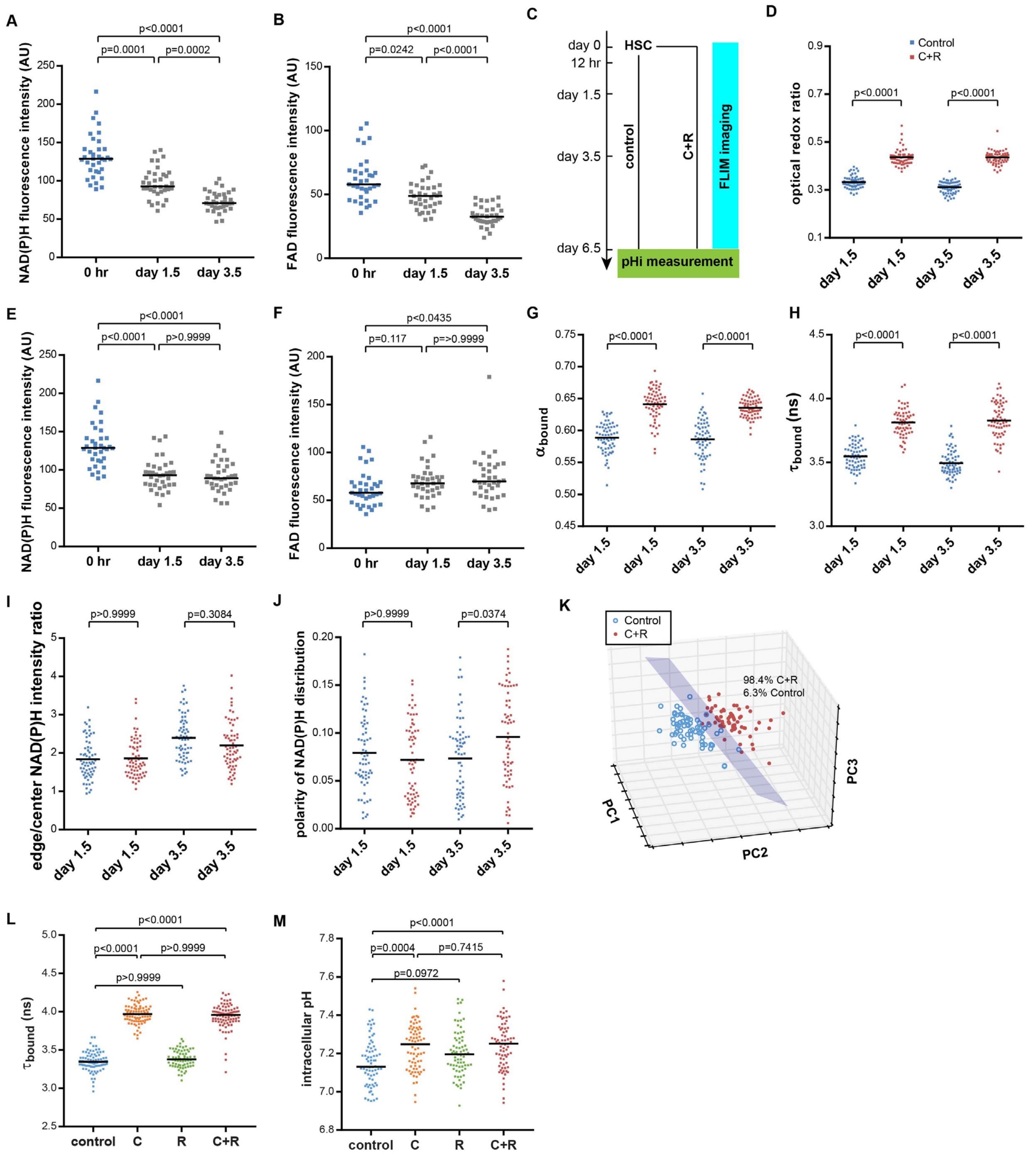

Figure S8. *Changes in optical metabolic characteristics during in vitro HSC cultures*. **Related to Figure 6**. (A) Changes of NAD(P)H fluorescence intensity and (B) FAD fluorescence intensity in normal cytokine culture. Dataset is the same as in **Figure 6B-G**. (C) Schematics of the experimental design with normal cytokine culture (control) and cytokine culture with the addition of CHIR99021+Rapamycin (C+R); (D) ORR in HSCs under C+R treatment and normal culture conditions; n=64 cells per condition per timepoint. (E) NAD(P)H and (F) FAD fluorescence intensity of HSCs with C+R treatment

at different time points; n = 35 cells per time point. (G) α_{bound}, (H) τ_{bound}, (I) edge/center ratio and (J) polarity of NAD(P)H intensity of HSCs under C+R treatment and normal culture conditions; n = 64 cells per condition per time point, from the same dataset as (D). (K) 3-D PCA analysis of cultured HSCs from C+R treatment and control conditions utilizing MOBs at day 1.5; n = 113 cells per condition. (L) Scatter plot of τ_{bound} at day 6.5 under different pharmacological treatment; n=71 cells per condition; (M) Scatter plot of pHi at day 6.5; n = 72 cells per condition; (A,B,D-J,L,M) Bars: Median. P-values: Kruskal-Wallis test.

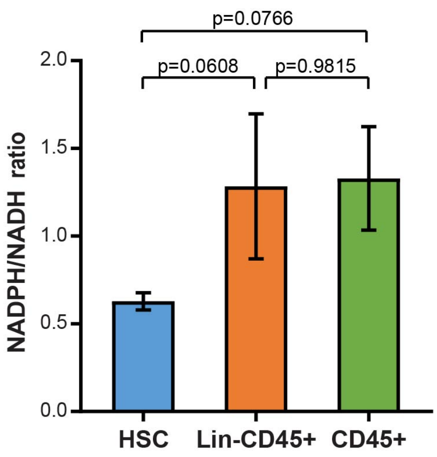

Figure S9. *HSCs have a lower NADPH/NADH ratio*. **Related to Figure 4**. Dataset is the same as in **Figure 4B**, n = 3 biological replicates. Error bars: SD. P-values: ordinary one-way ANOVA.

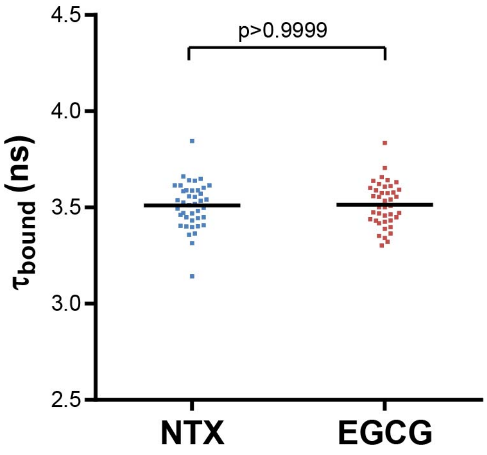

Figure S10. *Non-responsiveness of NAD(P)H τ_{bound} to EGCG treatment (1.5 hours) in HSCs.* **Related to Figure 2**. NTX: no treatment. Bars: Median. P value: Mann-Whitney test.

Supplemental Tables

Table S1. *Surface antigens used for sorting hematopoietic populations*. **Related to Figure 1 and Figure 5**.

Cell type	Surface antigens
HSC	*Lin- cKit+ Sca1+ Flk2- CD34- Slamf1+
MPP^{Flk2-}	Lin- cKit+ Sca1+ Flk2- CD34+
MPP^{Flk2+}	Lin- cKit+ Sca1+ Flk2+
CLP	Lin- IL7rα+ Flk2+
CMP	Lin- cKit+ Sca1- FcγR- CD34+
MEP	Lin- cKit+ Sca1- FcγR- CD34-
GMP	Lin- cKit+ Sca1- FcγR+
Lin-CD45+	Lin- CD45.2+
CD45+	CD45.2+
	*Lin (Lineage) markers include B220, CD3, CD4, CD8, Gr1, Mac1 and Ter119.

Table S2. *Antibodies used for sorting*. **Related to Figure 1 and Figure 5**.

Antigen	Conjugation	Vendor	Catalog#	Clone
B220	PerCP-Cy5.5	eBioscience	45-0452-82	6B2
CD3	PerCP-Cy5.5	eBioscience	45-0031-82	KT31.1
CD34	e660	eBioscience	50-0341-82	RAM34
CD4	PerCP-Cy5.5	eBioscience	45-0042-82	GK1.5
CD8	PerCP-Cy5.5	eBioscience	45-0081-82	53-6.7
cKit	APC-EF780	eBioscience	47-1171-82	2B8
FcγR	Biotin	eBioscience	13-0161-85	93
Flk2	PE-Cy5	eBioscience	15-1351-81	A2F-10
Gr1	PerCP-Cy5.5	Affymetrix	45-5931-80	8C5
IL7rα	PE-Cy7	BioLegend	135013	A7R34
CD45.2	A700	BioLegend	109822	104
CD45.1	PE-eFluor 610	Affymetrix	61-0453-82	A20
Mac1	PerCP-Cy5.5	eBioscience	45-0112-82	M1/70
Sca1	BV711	BioLegend	108131	D7
Slamf1	PE	BioLegend	115904	TC15-12F12.2
Biotin	PE-Cy5.5	Life technologies	SA1018	
Ter119	PerCP-Cy5.5	eBioscience	45-5921-82	Ter119

Transparent Methods

Fluorescence activated cell sorting

C57BL/6 mice were purchased from Jackson Laboratories. *Hoxb5*–tri-mCherry mice were from Dr. Irving Weissman's group at Stanford University. All mice bred at the Research Animal Facility of the University of Southern California. Animal procedures were approved by the Institutional Animal Care and Use Committee of the University of Southern California. Bone marrow cells were extracted from the crushed bones of 4–6 month-old C57BL/6 or *Hoxb5*–tri-mCherry mice with a mixed gender, and then immunostained for CD45+ and Lin-CD45+ cells, or enriched by cKit/IL7R and immunostained for HSCs and HPCs (**Fig. S1** and **Table S1, S2**). FACS sorting was carried out on a BD SORP FACSAria cell sorter at 4°C.

Fluorescence lifetime imaging

FACS-sorted murine cells were washed and resuspended in StemSpan™ SFEM II medium (STEMCELL Technologies) supplemented with 50 ng/mL stem cell factor (SCF) and 50 ng/mL thrombopoietin (TPO) (i.e., standard medium) at ~10^6/mL, and seeded in a 1536-well plate (Corning). For freshly isolated cells, the plate was then incubated at 37°C, 5% CO_2 for 1 hour to allow the cellular metabolism to reach a steady state before imaging. Fluorescence lifetime images were acquired with a Zeiss LSM-780 inverted microscope with a live cell work station (37°C, 5% CO_2) except otherwise indicated. Samples were excited at 740 nm in two-photon mode; the emission wavelength/optical filter was 460/80 nm for NAD(P)H and 540/50 nm for FAD. For image acquisition, the following settings were used: image size of 256 × 256 pixels (pixel size: 0.42 µm) and pixel dwelling of 12.41 µsec. For each picture, 20 frames were acquired and averaged. Each experiment was repeated at least 3 times.

Calculation of α_{bound}, τ_{bound}, and τ_{free}

To calculate the free and bound NAD(P)H lifetime in different cell types, we used a previously established 95% confidence ellipse method to determine their metabolic trajectories. In FLIM images, pixels from all the cells in the same image were pooled and plotted on the phasor plot. A 95% confidence ellipse was then generated and its long axis was utilized as the metabolic trajectory. τ_{bound} and τ_{free} were calculated as the intersections of the metabolic trajectory to the universal semicircle that represents the fluorescence lifetime values of the enzyme-bound and free co-enzymes with single-exponential decay (Stringari et al., 2012) (**Fig. 1B**). As initial experiments showed that τ_{free} is not significantly different between different cell types (**Fig. S2A**), we fixed the phasor position of τ_{free} (0.45 ns) for all the samples, and determined the τ_{bound} of the individual cells by extending the line from the coordinate of τ_{free} through the average (g, s) coordinate of the cell to the universal semicircle (Stringari et al., 2012) (**Fig. S2B**). Single-cell α_{bound} was calculated as the ratio of the distance of the cellular (g, s) coordinate to τ_{free} over the total length of the metabolic trajectory between τ_{bound} and τ_{free} on the phasor plot (**Fig. 1B**).

Chemiluminescent NAD(P)H assay

NADH/NADPH levels were measured using the NAD/NADH-Glo™ Assay (Promega, G9071) and NADP/NADPH-Glo™ Assay (Promega, G9081) kits, following the

manufacturer's protocols. Briefly, 10,000–30,000 cells were washed and resuspended in PBS, and then lysed by adding an equivalent volume of 0.2 N NaOH with 1% dodecyltrimethylammonium bromide (Sigma). The lysates were incubated at 60°C for 15 minutes, equilibrated to room temperature, and neutralized by 0.2 N HCl and 0.25 M Tris base. The samples were then added to a 384-well plate (Corning), mixed with the detection reagents and incubated at room temperature for 1 hour. The luminescence was detected by a Varioskan™ LUX multimode microplate reader (ThermoFisher Scientific). To eliminate the interference from the difference in the efficiency of the luminescence assays for NADH and NADPH, standard curves were generated using NADH (Sigma, N7410) and NADPH standards (Sigma, N8035) to convert the luminescence reading into the measured NADH and NADPH signals, respectively. The measured signals were then normalized to the cell number in each sample, which was assessed by FACS and verified by hemocytometer.

Intracellular pH measurement and calibration

To load the intracellular pH indicator, cells were incubated in standard medium with 10 μM SNARF-5F-AM (ThermoFisher Scientific) at 37°C for 30 minutes. Cells were then washed with PBS to remove the excess of SNARF-5F-AM and incubated at 37°C for an additional 1 hour to ensure de-esterification. Fluorescence excitation wavelength was 488 nm and the dual-peak emission was detected at 550/80 nm and 640/40 nm. The calibration of intracellular pH was carried out with the Intracellular pH Calibration Buffer Kit (ThermoFisher Scientific, P35379). *In vitro* cultured HSCs were first loaded with SNARF-5F-AM, washed with PBS, and then resuspended in pH 6.5 and 7.5 calibration buffers, which were supplemented with valinomycin and nigericin. Cells were imaged under the same imaging settings as above. A two-point pH calibration was performed to generate the standard curve. To study the influence of pH on the fluorescence lifetime of the enzyme-bound NAD(P)H, cultured HSCs without the pH indicator were washed with PBS, resuspended in the calibration buffers (pH 6.5 and 7.5) and imaged with FLIM. To study the influence of glucose concentration or 2DG on pHi, freshly isolated HSCs were seeded in DMEM (ThermoFisher Scientific) supplemented with different concentration of glucose or standard medium with/without 100mM 2DG. pHi measurement was carried out after FLIM imaging.

Drug inhibitions

For lactate dehydrogenase (LDH) and pyruvate dehydrogenase (PDH) inhibitions, freshly isolated HSCs, Lin-CD45+ and CD45+ cells from the same mouse were evenly split into three groups and resuspended in standard medium. LDH and PDH inhibition groups were then supplemented with 10 mM sodium oxamate (Cayman Chemical) and 0.2%wt 1-aminoethylphosphinic acid (AK Scientific, Inc.), respectively. Cells were then incubated at 37°C for 1 hour before imaging. For subcellular NAD(P)H distribution, the treatment group was supplemented with 200 nM rotenone (Sigma, R8875) to inhibit mitochondrial complex I and imaged with FLIM after 1 hour of incubation.

Single cell tracking and imaging

#1.5 glass-bottom petridishes were coated overnight with 5 μg/mL anti-CD43 (eBioR2/60, eBioscience) (Loeffler et al., 2018). Freshly isolated HSCs were seeded

and incubated at 37°C, 5% CO_2 for 1 hour to allow them to attach to the bottom of the coated petridish. Cells were then imaged in FLIM mode using a Leica SP8 FALCON inverted microscope with each cell's location recorded. Intracellular NAD(P)H and FAD were excited at 740 nm in two-photon mode; the emission wavelength/optical filter was 460/80 nm for NAD(P)H and 540/50 nm for FAD. FLIM images were acquired at the pixel size of 0.18 μm and pixel dwell time of 15.38 μs, with 8× line repetitions. For the single cell LDH inhibition study in **Fig. 2I and J,** 20× oxamate solution was prepared and injected in the petridish to reach a final concentration of 10 mM. After 1 h incubation, cells were FLIM-imaged again and the change of τ_{bound} before and after the treatment was tracked for each single cell. For the single-cell τ_{bound} and pHi correlation study (**Fig. S5A**), cells were FLIM-imaged, incubated with SNARF-5F-AM for 30 min, and imaged in standard confocal mode subsequently for the dual-peak emission at 550/80 nm and 640/40 nm under 488 nm excitation. During the whole imaging process, cells were incubated at 37°C with 5% CO_2.

Live-cell mitochondria staining and imaging
Freshly isolated HSCs (*Hoxb5*+ KLS) from *Hoxb5*–tri-mCherry mice were incubated with 50 μM Verapamil (Sigma, V4629) and 100nM Mitotracker Green FM (ThermoFisher Scientific, M7514) for 30 minutes. Cells were then washed with PBS and immobilized with CyGEL Sustain™ (Abcam, ab109205). Mitochondria and NAD(P)H were subsequently imaged under normal confocal mode (Ex: 488 nm, Em: 530/40 nm) and FLIM mode, respectively.

Identification of HSCs from KLS cells
A support vector machine (SVM) model with radial basis function kernel was trained with MOBs obtained from equal number of phenotypically defined HSCs (Lin-cKit+Sca1+Flk2-CD34-Slamf1+) and MPPs (Lin-cKit+Sca1+Flk2-CD34+ and Lin-cKit+Sca1+Flk2+). KLS cells sorted from *Hoxb5*–tri-mCherry mice were seeded in petridishes pre-coated with 5 μg/mL anti-CD43, and incubated at 37°C with 5% CO_2 for 1 hour before imaging. The mCherry signal was detected under standard confocal mode with a 580 nm excitation laser and a 620/40 nm emission filter. The same field of view was then imaged under FLIM mode, and the MOB parameters were calculated for each single cell. Each measured cell was predicted as an HSC or MPP using the SVM model, and the prediction was compared against the Hoxb5 expression (i.e. the ground truth data). The sensitivity and specificity of the model were then calculated. The python module "svm" from "scikit-learn" toolkit was utilized (Pedregosa et al., 2011).

Timelapse study on *in vitro* HSC culture and drug treatment
Sorted HSCs were suspended in standard medium supplemented with 1% penicillin and streptomycin (ThermoFisher Scientific), and seeded into 1536-well plate at ~1000 cells per 10 μL per well. For the drug-treatment groups, 3 μM CHIR99021 (Stemgent) and 5 nM rapamycin (Calbiochem) were added, as indicated (Huang et al., 2012). Cells were transferred to a 96-well plate (Corning) after 36 hours and cultured until the end of the experiment. Half of the medium in each well was changed at day 3 and 6.

Seahorse assay

Seahorse XF Glycolytic Rate Assay (Agilient) was carried out with cultured HSCs at day 6.5 with/without CHIR99021+rapamycin treatment. Glycolytic proton efflux rate (GlycoPER) was calculated from extracellular acidification rate (ECAR) and oxygen consumption rate (OCR) following the manufacturer's instruction.

Image analysis

All images were analyzed with a customized Python code. Background was subtracted based on intensity. For each pixel, phasor coordinates g and s were calculated based on the phase and modulation recorded (Stringari et al., 2012). For single-cell analysis, α_{bound} and τ_{bound} were calculated with the averaged g and s values. For subcellular analysis, the central region of individual cells was first isolated by binary erosion (iteration = 4 or 5, decided by cell size). Taking the central region as a continuum in terms of NAD(P)H distribution, its surrounding area with similar NAD(P)H intensity was then compensated into the "center". The rest of the cellular area was considered as the peripheral region (**Fig. 1H**). Autofluorescence intensity, α_{bound} and τ_{bound} were calculated. In **Fig. 1J, 5I** and **6H**, PCA was performed to reduce the dimension of datasets. LDA was then utilized to determine the gates that separate HSCs from other populations, or drug-treated cells from the control group in the 3-D PCA space.

Data plot and statistical analysis

All plots were made in Prism 7 (GraphPad), Python 2.7 (Python Software Foundation), and SimFCS 2.0 (Laboratory for Fluorescence Dynamics, University of California, Irvine). All data presented as the mean ± error had the error defined as 95% confidence interval (CI). All statistical analysis for single-cell scatter or box plots were performed with the Mann-Whitney (2 conditions) or Kruskal-Wallis tests (> 2 conditions) due to non-normal data distribution. All bars in the scatter plot are median, and box plots represent the 10th–90th percentile. All error bars in the bar graphs were plotted as standard deviation (SD) and compared with the Welch's t-test (2 conditions) or ordinary one-way analysis of variation (ANOVA) (> 2 conditions). Error bars in the population-level correlation x-y plots are the standard error of the mean (SEM). The linear regressions with p-values were tested with zero-slope hypothesis. P-values are indicated as numbers (* $p < 0.05$, ** $p < 0.01$, *** $p < 0.00$, and **** $p < 0.0001$) or as n.s. (not significant, $p > 0.05$) on the plots.

Supplemental References

Huang, J., Nguyen-McCarty, M., Hexner, E.O., Danet-Desnoyers, G., and Klein, P.S. (2012). Maintenance of hematopoietic stem cells through regulation of Wnt and mTOR pathways. Nat Med *18*, 1778-1785.

Loeffler, D., Wang, W., Hopf, A., Hilsenbeck, O., Bourgine, P.E., Rudolf, F., Martin, I., and Schroeder, T. (2018). Mouse and human HSPC immobilization in liquid culture by CD43- or CD44-antibody coating. Blood *131*, 1425-1429.

Pedregosa, F., Varoquaux, G., Gramfort, A., Michel, V., Thirion, B., Grisel, O., Blondel, M., Prettenhofer, P., Weiss, R., Dubourg, V.*, et al.* (2011). Scikit-learn: Machine learning in python. Journal of Machine Learning Research *12*, 2826-2830.

Stringari, C., Edwards, R.A., Pate, K.T., Waterman, M.L., Donovan, P.J., and Gratton, E. (2012). Metabolic trajectory of cellular differentiation in small intestine by Phasor Fluorescence Lifetime Microscopy of NADH. Sci Rep *2*.
